# New Perspectives on Host-Parasite Interplay by Comparative Transcriptomic and Proteomic Analyses of Schistosoma japonicum

**DOI:** 10.1371/journal.ppat.0020029

**Published:** 2006-04-14

**Authors:** Feng Liu, Jiong Lu, Wei Hu, Sheng-Yue Wang, Shu-Jian Cui, Ming Chi, Qing Yan, Xin-Rong Wang, Huai-Dong Song, Xue-Nian Xu, Ju-Jun Wang, Xiang-Lin Zhang, Xin Zhang, Zhi-Qin Wang, Chun-Liang Xue, Paul J Brindley, Donald P McManus, Peng-Yuan Yang, Zheng Feng, Zhu Chen, Ze-Guang Han

**Affiliations:** 1 Chinese National Human Genome Center at Shanghai, Shanghai, China; 2 National Institute of Parasitic Diseases, Chinese Center for Disease Control and Prevention, Shanghai, China; 3 State Key Laboratory of Medical Genomics, Shanghai Second Medical University, Shanghai, China; 4 Department of Parasitology, Shanghai Second Medical University, Shanghai, China; 5 Department of Tropical Medicine, Tulane University Health Sciences Center, New Orleans, Louisiana, United States of America; 6 Queensland Institute of Medical Research and Australian Center for International Health and Nutrition, Brisbane, Queensland, Australia; 7 Proteomic Center and Department of Chemistry, Fudan University, Shanghai, China; University of Edinburgh, United Kingdom

## Abstract

Schistosomiasis remains a serious public health problem with an estimated 200 million people infected in 76 countries. Here we isolated ~ 8,400 potential protein-encoding cDNA contigs from Schistosoma japonicum after sequencing circa 84,000 expressed sequence tags. In tandem, we undertook a high-throughput proteomics approach to characterize the protein expression profiles of a number of developmental stages (cercariae, hepatic schistosomula, female and male adults, eggs, and miracidia) and tissues at the host-parasite interface (eggshell and tegument) by interrogating the protein database deduced from the contigs. Comparative analysis of these transcriptomic and proteomic data, the latter including 3,260 proteins with putative identities, revealed differential expression of genes among the various developmental stages and sexes of S. japonicum and localization of putative secretory and membrane antigens, enzymes, and other gene products on the adult tegument and eggshell, many of which displayed genetic polymorphisms. Numerous S. japonicum genes exhibited high levels of identity with those of their mammalian hosts, whereas many others appeared to be conserved only across the genus *Schistosoma* or Phylum Platyhelminthes. These findings are expected to provide new insights into the pathophysiology of schistosomiasis and for the development of improved interventions for disease control and will facilitate a more fundamental understanding of schistosome biology, evolution, and the host-parasite interplay.

## Synopsis

Schistosomiasis remains a major public health problem in the developing world. *Schistosoma japonicum,* the Oriental blood fluke, causes intestinal schistosomiasis in China and the Philippines. Knowledge of the genome and proteome of this worm should improve understanding of biomedical aspects of schistosomiasis. This study represents the first major attempt to characterize the majority of the expressed genes and proteins of a human blood fluke through rigorous, high-throughput genomic and proteomic methodologies. The findings of this study provide a unique resource of numerous schistosome genes and information on protein profiles of the different developmental stages of *S. japonicum.* Many of the newly discovered proteins are localized on the surface of the worm and its eggs, and they are likely to be involved in the pathogenesis of schistosomiasis. Furthermore, genetic variants found in many of these new genes likely reflect the ability of this important human pathogen to adapt and respond to environmental pressures and the capacity of the parasite to respond to anti-schistosomal therapies. Comparison of these S. japonicum genes with those from mammals and other organisms will facilitate advances in the understanding of blood fluke biology and evolution.

## Introduction

Schistosomiasis remains one of the most prevalent and serious of the parasitic diseases, with an estimated 200 million people infected in 76 countries and territories, located predominantly in tropical and subtropical regions. The disease is caused by three major schistosome species, *Schistosoma japonicum, Schistosoma mansoni,* and Schistosoma haematobium [1]. Schistosomes have a complex life cycle with specific, differential gene expression for adaptation to their intermediate, snail, and definitive mammalian host environments. Eggs deposited by the adult female schistosomes embolize in the liver, intestines, and other sites and represent the key contributor to the pathology and morbidity associated with schistosomiasis. The highly adapted relationship between schistosomes and their mammalian hosts appears to involve parasite exploitation of host endocrine and immune signals [2–4]. Evasion strategies that underpin avoidance of the host immune system, allowing schistosomes to survive for years despite strong host immunological responses, have long confounded and intrigued investigators intent on controlling these parasites through development of an effective vaccine. A comprehensive deciphering of the schistosome genome, transcriptome, and proteome has become increasingly central for understanding the complex parasite-host interplay and for delivering candidate drug and vaccine targets [5,6]. Although many tens of thousands of expressed sequence tags (ESTs) derived from S. japonicum and S. mansoni were recently released to GenBank by us and others [7,8], and whereas ESTs are useful for cataloging expressed genes, these ESTs are not the ideal format of genetic information to study gene function because many provide only partial sequence coverage of the matching gene. Accordingly, genome-scale collections of the full-length cDNAs with potential coding sequences (CDSs) of expressed genes have become important for the analysis of the structure and function of the estimated 14,000–20,000 genes of the schistosome parasite [8,9]. Furthermore, global proteomics analyses offer a unique means for determining not only protein identification for genome annotation but also for subcellular localization of these proteins. In this present report, we describe the isolation of ~ 8,420 potential protein-coding RNAs from *S. japonicum.* Moreover, in tandem with nucleotide sequence analysis of the CDSs, we undertook high-throughput proteomics analyses, involving high resolution in-line two-dimensional-nano-liquid chromatography (2D-nano-LC) and tandem mass spectrometry (MS/MS) analysis to characterize gene and protein expression within different developmental stages of the schistosome (cercariae, hepatic schistosomula, adults, eggs, and miracidia), as well as in tegumental preparations and the eggshell. We interrogated the protein and peptide datasets deduced from these CDSs (Figure S1). We anticipate that the findings from these comparative genomic, transcriptomic, and proteomic analyses should lead to a more profound understanding of schistosome biology, the host-parasite relationship, molecular mechanisms of immunopathology of schistosomiasis, and the development of innovative intervention strategies.

## Results

### The Transcriptome of *S. japonicum*

Previously, we reported an initial analysis of the transcriptomes of adult and egg stages of S. japonicum from 43,707 5′ ESTs that we assigned to 13,131 gene clusters, of which 2,706 clusters were similar to known proteins deposited in GenBank (BLASTP cutoff of E value 10^−20^), and of which 611 genes containing complete CDSs were isolated [7]. To isolate more full-length cDNAs with complete or partial CDSs of S. japonicum genes, we constructed new cDNA libraries with longer inserts (mean size ~ 2 kb) from mixed-sex adult worms and hepatic schistosomula. Long-read sequencing of both 5′ and 3′ ends was subsequently performed on clones randomly selected from the new cDNA libraries. Representative clones of assembled sequences with potential CDSs were also sequenced. A total of 98,770 raw ESTs derived from 79,639 clones, which were composed of 55,063 new ESTs and the 43,707 ESTs reported previously [7], were quality-trimmed using Phred 20 after removing repetitive, mitochondrial, ambiguous, and vector sequences (Table S1). The available 84,449 clean ESTs, including 67,383 (79.9%) 5′ ESTs and 17,026 (20.1%) new 3′ ESTs, were assembled into 14,962 clusters with an average length of 783 base pairs (bp) (Table S1) (10,518 contigs and 4,444 singletons), where the length of gene clusters was extended and the quality was significantly improved as compared to our earlier report [7]. To independently evaluate the cluster redundancy and gene coverage of this expanded transcriptomic dataset, we collected 213 S. japonicum mRNAs deposited in GenBank prior to the large-scale EST sequencing project (before December, 2002). After removing retrotransposon and ribosomal sequences, the remaining 172 CDSs, of which 105 encode complete CDSs and 67 have partial coding sequences, may represent 126 distinct genes. Of these 126 genes, 110 (87.3%) could be found in the new expanded S. japonicum EST dataset, whereas 92 (73%) were represented by 159 of the new 8,420 clusters with potential CDSs, where an average of 1.73 clusters was assigned into a gene. This analysis indicated that the majority of S. japonicum genes may be present in the expanded transcriptome dataset.

Of these 14,962 clusters, 8,420 (56.3%) appeared to have potential CDSs according to similarity, protein identification, or length (hypothetical genes encoding at least 100 amino acids) (Figure S1). Specifically, of these 8,420 clusters with CDSs, 3,077 (36.5%) were included with apparently complete CDSs, whereas 3,695 (43.9%) were considered to have partial CDSs lacking the start or stop codons, although these CDSs exhibited identity to known proteins from other organisms (BLASTP cutoff of E value 10^−20^). The remaining 1,648 (19.6%) clusters were considered to be genes encoding hypothetical proteins of unknown function. The average length of the 3,077 genes with entire CDSs, including the 611 genes reported previously [7], was 1,024 bp; within these genes, the average length of the CDS was 606 bp, encoding 202 deduced amino acid residues (Figure S2). The majority (2,112; 68.6%) of these entire CDSs could encode proteins with lengths ranging from 100 to 300 amino acid residues.

**Figure 1:**
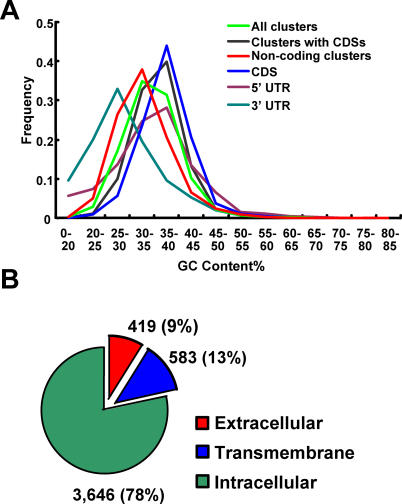
Characteristics of S. japonicum Clusters with Potential CDSs (A) A comparison of GC content among all clusters, clusters with protein-coding genes, non-coding clusters, protein-coding regions, and 5′ and 3′ UTRs. (B) The subcellular localization of putative proteins deduced from complete CDSs and hypothetical genes predicted with the bioinformatics tools SignalP, TMHMM, and PSORT II.

To characterize the transcriptomic information, we further analyzed the guanine-cytosine (GC) content of those clusters with or without CDSs (Figure 1A). The GC content of all 14,962 clusters ranged broadly between 10% and 55% with a significant peak at ~ 32%, whereas the prominent peak of GC content of the clusters with CDSs, in particular protein-coding regions, was apparent at ~ 37% GC. By contrast, the GC content of the 3′ UTRs was obviously reduced, with a peak at only ~ 27%. Furthermore, 419 (8.9%) and 583 (13.6%) of 4,725 complete and hypothetical CDSs were predicted as secretory and membrane proteins, respectively (Figure 1B and Table S2).

To explore the biological characteristics of *S. japonicum,* the 5,077 CDSs with identity to known proteins (BLASTP E value of 10^−10^), were further categorized based on Gene Ontology (GO) (see Materials and Methods for details and http://function.chgc.sh.cn/sj-proteome/index.htm). We assigned 873 (17.2%) to 12 main molecular functional categories and 829 (16.3%) to 15 main biological process categories. Similarly, among 2,984 CDSs encoding InterPro protein domains (Tables S2 and S3), 842 (28.2%) and 894 (30%) were assigned into molecular functional and biological process categories, respectively (Figure S3 and Table S4).

### A Proteomic View of the S. japonicum Life Cycle

We employed 2D-nano-LC, one-dimensional PAGE, and MS/MS in a shotgun proteomics approach to further profile the protein expression of various developmental cycle stages of S. japonicum (including cercariae, hepatic schistosomula [whole worms and their tegument], adults [mixed-sex, females, males, and their teguments], eggs [intact eggs and empty eggshells], and miracidia). This was accomplished by interrogation of the combined human, mouse, and rabbit mammalian host protein and peptide databases and comparison with *S. japonicum,* which we had assembled from the newly obtained transcriptomic data (Figure S1 and Protocol S1), as previously described [10].

**Figure 2:**
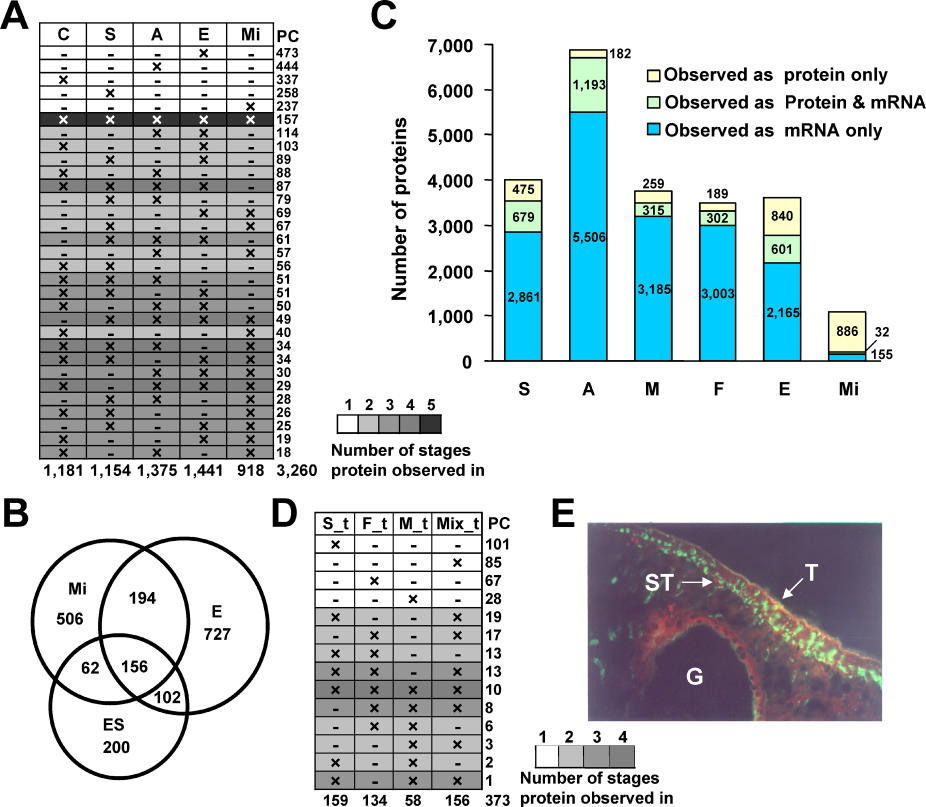
A Comprehensive Proteomic Survey of S. japonicum (A) Protein expression profiling across the life cycle, including C, cercariae; S, hepatic schistosomula; A, adults; E, eggs; and Mi, miracidia. The numbers on the right side represent the number of proteins observed at the developmental stages indicated to the left. The icon at the bottom indicates the expression pattern during the life cycle, and the numbers indicate the amount of stages in which protein is observed. PC, protein count. (B) Venn diagram of the distribution of observed proteins among E, eggs; Mi, miracidia; and ES, eggshell-containing samples. (C) Comparison of transcriptomic and proteomic data of the developmental stages. (D) Protein expression profiling of tegumental proteins across the life cycles, including S_t , hepatic schistosomula; F_t, female; M_t, male; and Mix_t, mixed-sex adults. (E) The tegumental localization of SjFKBP50 (immunophilin) by immunofluorescence microscopy. T, tegument; ST, subtegument; G, gut.

**Figure 3:**
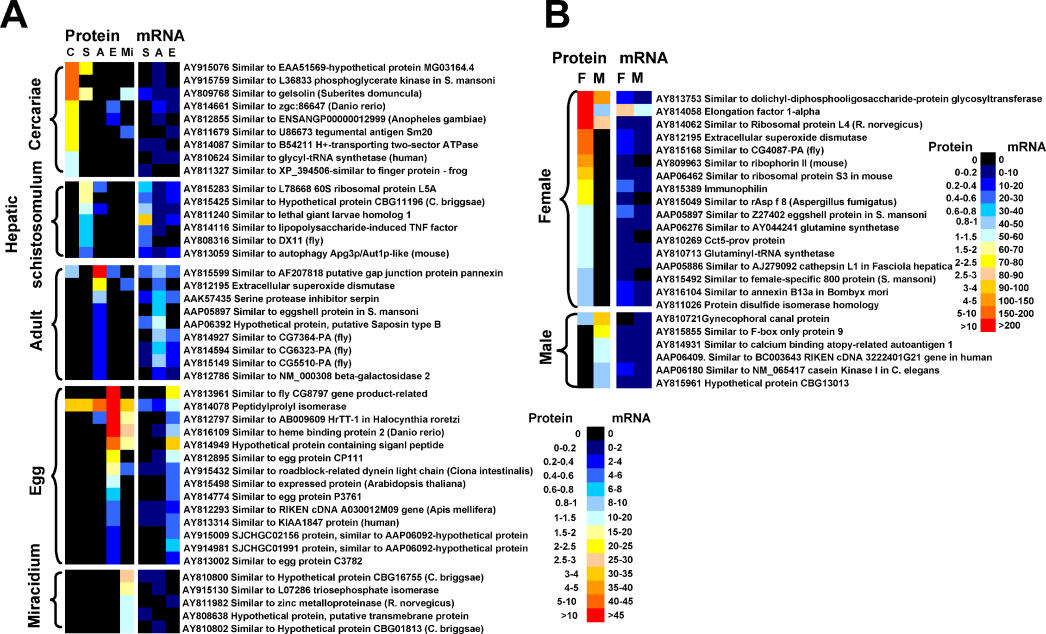
Correlation between Transcriptomic and Proteomic Data of Selected Schistosome Proteins among Developmental Stages and Sexes (A) Some developmental stage-enriched proteins were consistent with the transcriptomic data indicated. C, cercariae; S, hepatic schistosomula; A, adults; E, eggs; and Mi, miracidia. Those significant proteins with more than a 3-fold difference were consistent with the differential display (*p* < 0.05) based on EST copy numbers throughout hepatic schistosomula, adults and eggs, or between female and male worms. For cercariae and miracidia, we only showed some proteins with more than 3-fold differences throughout the life cycle based on quantitative proteomics since both stages are devoid of ESTs. However, the selected proteins have low EST copy numbers in other developmental stages. (B) Gender-enriched proteins are also shown as colored boxes: F, females; M, males. Protein abundance and EST frequencies are shown in different colored boxes, as the icons indicate where the EST frequency represents the ratio of EST copy numbers of a given gene to the total number of all ESTs derived from the corresponding cDNA library and then multiplied by 10,000. The full datasets of stage-enriched and gender-enriched proteins with consistency between transcriptomic and proteomic data were given in Figure S4.

More than 420,000 highly accurate MS/MS spectra, generated from more than 400 protein sub-fractions from all of the discrete samples, were searched against the combined host-parasite protein database using probability-based scoring. The resulting findings indicated that a total of 26,484 distinct peptides with significant probability scores could be confidently assigned into 3,260 unique proteins that accounted for 38.7% of the 8,420 CDSs. Of these unique proteins, 1,181, 1,154, 1,375, 1,441, and 918 were identified from cercariae, hepatic schistosomula, adults, eggs, and miracidia, respectively (Figure 2A and Table S2). There were 337, 258, 444, 473, and 237 proteins that were identified in one or another of these developmental stages only; suggesting that, despite the fact that the GO classification displays little discrimination among the different developmental stages (Table S2), certain proteins are stage-enriched or are expressed in response to different environmental stimuli. However, some proteins identified in only one stage by the proteomics analysis had ESTs in more than one stage, as did some gender-enriched proteins. This apparent inconsistency may reflect the incompleteness of the proteomic dataset due to limitations in sensitivity of the proteomic technologies that we employed. Also, some transcripts may be relatively stable and might persist through several stages but be translated in a much shorter window, contributing to the discrepancy between the proteomic and transcriptomic data. Only those proteins with more than 3-fold differences throughout the life cycle or between the female and male worms based on quantitative proteomics [11], and which were consistent with the transcriptome data, are highlighted in Figures 3 and S4. Interestingly, sex-enriched expression was far more dramatic than stage-enriched expression. Herein, the correlation between transcriptomic and proteomic data (*p* < 0.05) (based on EST copy numbers using tools available at http://www.igs.cnrs-mrs.fr/~audic/significance.html) [12] among the three developmental stages, including hepatic schistosomula, adults, and eggs, or between female and male worms, was considered to be significant. For cercariae and miracidia, we only show some proteins with more than 3-fold differences throughout the life cycle based on quantitative proteomics, since only a small number of ESTs were available for these two larval stages that were insufficient to statistic analysis.

Putative deoxyribodipyrimidine photo-lyase (DNA photolyase) was found only in cercariae (the mammalian-infective, larval form), implying that the free-living cercariae might catalyze the light-dependent monomerization of cyclobutyl pyrimidine dimers as a photo-reactivating enzyme, upon exposure to ultraviolet radiation [13]. The stage-specific protein SPO-1, which is preferentially expressed in schistosome sporocysts within their snail hosts [14], and putative sex-determining region Y protein (SRY), which is a testis-determining factor [15], were all expressed in cercariae, indicating that this stage undergoes significant development associated with male sex determination (Table S2). In addition, proteins similar to a number of well-known receptors, including vasopressin-activated calcium-mobilizing receptor (cullin 5), dioxin receptor, and tri-spanning orphan receptor were expressed in cercariae (although not only in this stage), suggesting that these larvae may have evolved specific molecular processes for detecting their mammalian hosts. However, an ortholog of S. mansoni cercarial elastase, a major enzyme involved in skin penetration, has not been identified to date from S. japonicum [16].

A number of proteins with identity to homologs, known in other species to be associated with neural development, including ubiquitin-protein ligase NEDD4-like, prion protein interacting protein, karyopherin (which facilitates nuclear import), budding uninhibited by benzimidazoles1 (a kinase involved in spindle checkpoint function), and Su (var) 3–9 (the major heterochromatin-specific HMTase) were highly expressed in hepatic schistosomula. Their presence may reflect the complex physiology of these immature adult worms, which are adapting to dramatic environmental changes as they migrate from the liver to the mesenteric veins of the intestines, which is the preferred site of the adult stage. The hepatic schistosomula also expressed numerous enzymes associated with digestion of hemoglobin [17,18], which reflects the nutritional dependence of this stage (and mature adult worms) for ingested host red blood cells.

Of the 1,375 proteins identified from adult worms, 491, 574, and 723 were identified from females, males, and mixed adults, respectively; of these, 444 proteins were identified only in adults (Figure 2A and Table S2). In addition to cytoskeleton and motor proteins, chaperones, extracellular matrix molecules, and enzymes associated with digestion of haemoglobin, a number of proteins similar to known proteins associated with developmental and sexual maturation, including forebrain embryonic zinc-finger like (Fezl), histone H2A (gonadal), and eggshell protein were identified, and they may play key roles in schistosome growth and sexual maturation (Figures 3A and S4, Table S2). Putative ribophorin II, extracellular superoxide dismutase, and female-specific 800 protein appeared to be preferentially expressed in adult females; whereas gynecophoral canal protein, F-box only protein 9, and amidase were preferentially expressed in males (Figures 3B and S4, Table S2). The gynecophoral canal protein has been shown previously to be localized to the gynecophoral canal of S. mansoni male worms [19].

It is noteworthy that putative C1-tetrahydrofolate synthase was also identified in adults and hepatic schistosomula, suggesting that folic acid and its derivatives could be critical for growth, development, and differentiation, as well as for normal cellular function. However, other folic acid pathway elements, e.g., dihydrofolate reductase, which has been characterized in flatworms [20,21], were not found in this study.

Schistosome eggs are directly responsible for granuloma formation in the liver and are the major cause of pathology in schistosomiasis. It is noteworthy that calcium influx could be important for eggs because several Ca^2+^-associated polypeptides, including high voltage-activated calcium channel (beta subunit 2) and calcium/calmodulin-dependent protein kinase II (delta isoform 3) were found only in eggs [22]. Additional proteins that could be involved in development, including twister, nocturnin (a circadian clock-regulated gene), craniofacial development protein 1, and transducin-like enhancer of split 3 (TLE3) were highly expressed in eggs. Furthermore, many molecules involved in mitosis, including microtubule-associated protein and regulator of G-protein signaling 2, were also expressed in eggs; this suggests that a small proportion of miracidia would still be maturing within eggs newly laid by female adults, although the majority of eggs deposited in the liver would already be fully developed and quiescent.

Of 918 proteins identified from newly hatched miracidia, 412 (44.7%) were also located in eggs, while 237 were found only in miracidia (Figure 2A and 2B). Along with several motor proteins, some receptor-like proteins, and related proteins involved in neural development, including Notch receptor, GABA receptor, dioxin receptor, and acetylcholine receptor (alpha-3 chain) were expressed in this developmental stage (Table S2). This implies that the free-living, motile miracidium can accept external signaling molecules from the snail intermediate host through receptors linked to the miracidial nervous system, in addition to being able to respond to internally produced (self) signals; although supporting functional evidence is currently not yet available.

### Comparison of the S. japonicum Transcriptome and Proteome

To further investigate the relationship between the transcriptome and proteome of *S. japonicum,* we compared the available ESTs and proteins identified in the various developmental stages examined in this study. Of 18,579 ESTs representing 3,540 potential CDSs derived from hepatic schistosomula, more than half, 679 (58.8%) of 1,154 proteins identified, were consistent with the transcriptomic data for this developmental stage (Figure 2C). Similarly, 41.7%, 54.9%, and 61.5% of the proteins identified in egg and male and female worms were consistent with the transcripts, respectively. In the adult stage samples, 1,193 (86.8%) of 1,375 appeared to have transcripts in 6,699 CDSs assigned from 52,742 ESTs from adult stages, exhibiting the highest overlap between the proteomic and transcriptomic data for the developmental stages investigated here, suggesting that a large amount of transcriptomic data could be helpful to the annotation of proteomic resource in addition to the protein identification. The lowest level (3.5%) of concordance between the transcriptomic and proteomic datasets was obtained with the miracidium, and this finding probably reflects the small number of ESTs available for this stage. Nonetheless, the overall findings indicated that tandem proteomic and transcriptomic approaches will provide distinct, yet complementary, views in profiling gene expression in discrete schistosome developmental stages, in like fashion to the situation reported for *Plasmodium* [23].

### Tegument and Eggshell Proteins

The surface tegument that covers the schistosomulum and adult stages of schistosomes contributes centrally to host-parasite interactions, being critical for nutrient uptake, parasite growth, and development and as a protective barrier against host immune responses. In light of their importance for parasite survival, tegumental proteins are recognized as prime candidate targets for chemotherapy and immunotherapy of schistosomiasis [24]. We prepared the tegumental samples according to an established method [25] and identified 373 tegumental proteins—134 from adult females, 58 from adult males, 156 from mixed-sex adults, and 159 from hepatic schistosomula (Figure 2D and Table S2). It is noteworthy that 85 tegument proteins were only found in mixed-sex adults, which might reflect heterogeneity in protein extraction and peptide detection with the mass spectrometer for different tegumental batch samples. Among the tegument proteins in our preparations, several cytoskeleton and motor proteins (actins, tubulins, paramyosin, tropomyosin, myosin, and dynein light chain 1) [23], 22.6 kDa tegument membrane-associated antigen [26], tegumental antigen Sm20 [27], glutathione-S-transferase [28], nitric oxide synthase 1 (NOS1) [29], leucine aminopeptidase [30], 14–3–3 proteins [31], and SnaK [32], as well as a cathepsin B-like cysteine protease precursor [33] and 21.7 kDa antigen [34] have been characterized previously as tegumental proteins.

Many chaperones, including heat-shock proteins (60, 70, 86, and 90 kDa) and chaperonins, were also identified in the tegument samples. Further, it is possible other extracellular matrix proteins, transporters, and membrane proteins that were located in the tegumental protein assemblage, including collagen (type I, alpha 3), annexins, osteonectin (SPARC), and presenilin may have roles in the host-parasite interplay at the schistosome surface. Several enzymes involved in redox homeostasis, such as antioxidative thioredoxin peroxidases and manganese superoxide dismutase were situated in the tegument, implying that this layer could provide protection against therapeutic drugs, environmental toxins, and products of oxidative stress through detoxification pathways. Interestingly, we did not detect glutathione-S-transferase among the tegumental proteins in our samples. Components of Ca^2+^ ion signaling pathways, including calpain, calreticulin, and calcineurin A were expressed in the tegument, suggesting that these pathways could play an important role in the maintenance of tegumental functions, and thus the key components in the pathways might be considered as putative drug targets [35,36]. Furthermore, to evaluate the proteomic data, an immunofluorescence assay was employed to confirm the localization of immunophilin (FK506-binding protein 50) identified as a tegumental protein, using a monoclonal antibody prepared against recombinant S. japonicum immunophilin. The assay indicated that immunophilin was limitedly localized in the subtegumental region (Figure 2E), directly supportive of the proteomics findings.

The schistosome eggshell is sclero-proteinaceous in nature and is lined internally by a vitelline membrane within which the miracidium develops [37]. A mechanism for eggshell production in S. mansoni has been proposed [22]. In the present study, the eggshell-containing samples were collected for proteomic identification soon after the miracidia had hatched from the eggs. Of 520 proteins found in the eggshell preparations, 258 and 218 were also located in samples from intact eggs and from miracidia, respectively (Figure 2B). Several proteins with similarity to known eggshell proteins, including p48 eggshell protein [38] and thioredoxin peroxidase [39], as well as previously characterized egg proteins, such as p40 major egg antigen, 21.7 kDa, and SM22.6 antigens (A12) were identified in the eggshell sample. Additionally, many motor proteins and chaperones were found in the eggshell. It is noteworthy that some enzymes or proteins involved in redox homeostasis were also found in the eggshell-containing sample, and this suggests that like the tegument, the eggshell could provide a protective biochemical barrier to oxidative stress. It is generally accepted that antigens released by the miracidium within the egg are responsible for the onset of granuloma formation around the egg, leading to disease [40]. Interestingly, immunity-associated and cell adhesion-related proteins, such as endoplasmin (gp96), immunophilin (FK506-binding protein 50), HLA-B associated transcript 1, and platelet glycoprotein IIIa (GPIIIa) were identified in the eggshell sample. These antigens may be contributing to the molecular mechanisms associated with granuloma formation. For example, endoplasmin (gp96), a known inflammatory mediator, not only promotes CD8+ and CD4+ T cell effector functions, as a specific co-stimulatory molecule [41,42], but also activates dendritic cells, neutrophils, or monocytes and promotes phagocytosis [43]. Like the tegument, the eggshell also contains proteins involved in calcium flux pathways, as well as signaling molecules such as 14–3–3 proteins.

### Genetic Polymorphisms

**Figure 4:**
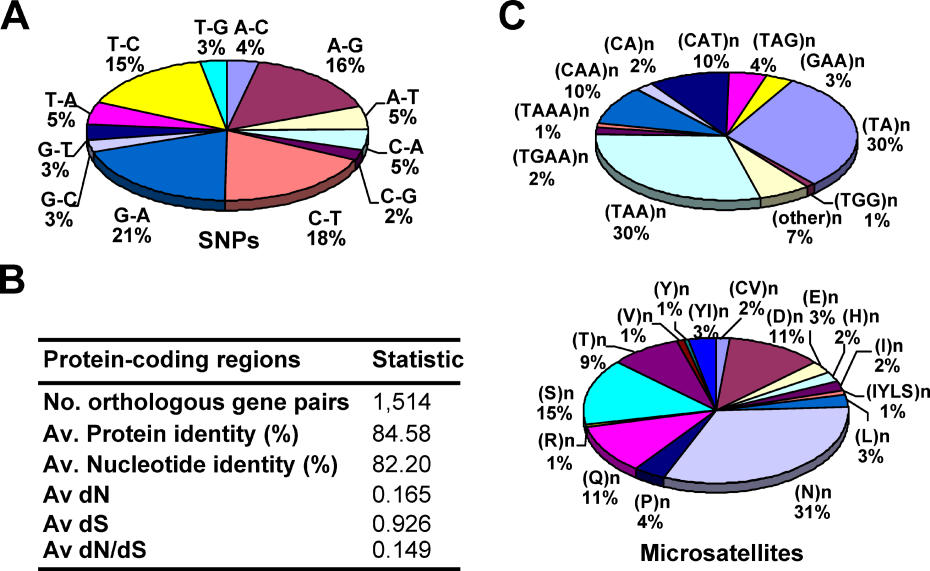
Genetic Polymorphisms in S. japonicum Genes (A) The distribution of nucleotide transition and transversion due to SNPs. The bars between single-letter nucleotides indicate the substitution of the latter base for the former. (B) Summary of the dN/dS analysis. The average dN/dS ratios and identities of coding regions are shown for all orthologous genes between S. japonicum and S. mansoni. Av, average. (C) The pattern distributions of the repeated amino acid residues deduced from microsatellite repeats within protein-encoding regions.

Genetic variation in schistosome populations can be expected to contribute to differences in infectivity, development in intermediate and definitive hosts, drug sensitivity, pathogenicity, and immunogenicity. In the present study, about 13,000 cercariae from naturally infected snail populations from Anhui Province, China were employed to experimentally infect laboratory mice in order to obtain the schistosome samples that we investigated in the transcriptomic analyses. Within 5,267 contigs with at least four ESTs (the minimum required for redundancy-based single nucleotide polymorphism [SNP] detection) [44], we could identify 7,286 SNP sites, including 6,038 in the cluster with CDS and the reminding 1,248 in non-protein coding clusters, with a redundancy of two or more ESTs in 1,812 (21.5%) contigs with an average SNP density of 1/288 bp, according to stringent criteria [44,45] (Tables S5 and S6). Of these 1,812 contigs, 1,496 contained potential CDSs, whereas 316 did not. Interestingly, of 6,038 SNP sites occurring in the 1,496 genes with CDSs, 3,673 were localized in protein-encoding regions of 1,121 genes with a SNP density of 1/244 bp; 521 were found in the 5′ UTRs of 270 contigs with a density of 1/133 bp and 1,844 in the 3′ UTRs of 625 contigs with a density of 1/158 bp, indicating that the protein-encoding regions of the S. japonicum genome display lower SNP density compared to those of UTRs. In these SNPs, the transition of C-T/T-C and A-G/G-A was found in 33% and 37% of SNPs, respectively, whereas transversions led by SNPs accounted for 30% (Figure 4A). Moreover, 2,272 (61.8%) of 3,824 SNPs in 3,673 sites could only induce synonymous substitutions in coding regions, whereas 1,552 (40.6%) nonsynonymous SNPs may lead to protein variations in 601 CDSs. Furthermore, a small number of the SNPs may abrogate or introduce stop codons to cause an extension or truncation.

To evaluate the potential significance of the genes with potential nonsynonymous SNPs within *S. japonicum,* and to begin to investigate evolutionary pressures acting on schistosome genes, we first calculated the ratio of nonsynonymous to synonymous substitutions (dN/dS) between 1,514 orthologs found in S. japonicum and *S. mansoni.* In general, it appeared that the orthologous gene pairs were under purifying selection pressure due to low average dN/dS ratios (0.149) (Figure 4B). Interestingly, of the 601 CDSs with nonsynonymous SNPs, 185 (93.0%) of 199 with detectable orthologs in S. mansoni had a higher than average dN/dS ratio value (Table S6), including *putative ribosome-associated protein P40* (3.125), *protein disulfide isomerase* (0.694), *21.7 kDa antigen* (0.524), and *immunophilin* (0.457). This may endow the schistosome population with the potential for adaptation to environmental niches under diverse selection pressures, including host immune responses. Moreover, 335 of the 601 CDSs detected by the proteomics analysis, including 72 tegument-localized proteins such as paramyosin, fimbrin, and prosaposin, as well as 89 eggshell proteins such as antigen SM22.6 (A12), calcium-binding protein Sj66, G protein alpha subunit, and flavoprotein (Fp) may display antigenic polymorphisms due to the candidate SNPs. The SNPs appear to represent a capacity of the schistosome population to parry, modify, or attenuate host immunological responses during infection of the mammalian host.

To estimate the potential SNPs representing either polymorphisms between chromosomal homologs or polymorphisms between individuals, we performed genomic DNA sequencing on PCR-amplified products from 30 individual worms for three genes with one or two potential SNP sites each, a total of four SNP sites. This revealed that the homogeneous 62 (83.8%) of all available 74 sequences exhibited differences at all four sites between individuals, with the remaining 12 (16.2%) sequences showing heterogeneous peaks at three sites within single individuals, suggesting that a small proportion of this polymorphism data as “background” variation could be due to differences between both alleles of the same gene.

**Figure 5:**
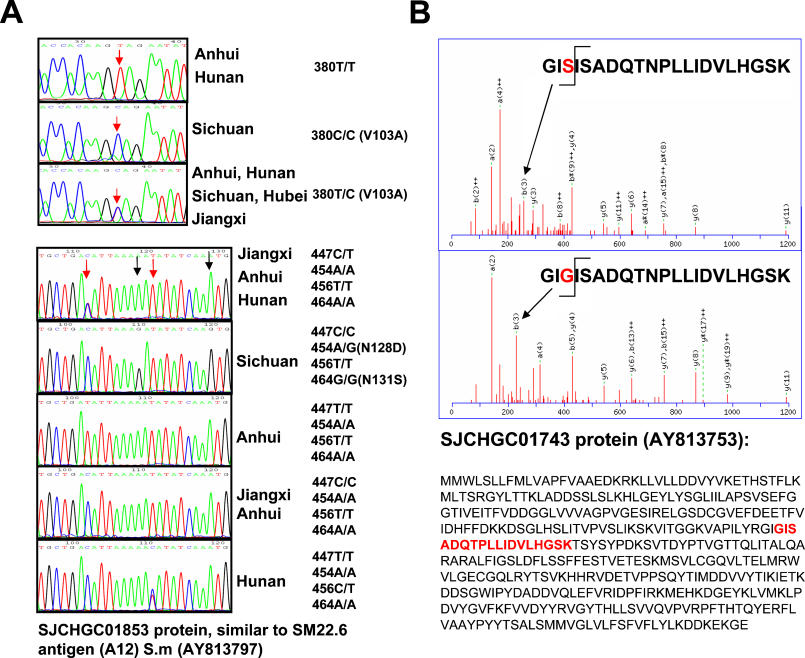
Validation of Potential SNPs by RT-PCR and MS/MS Spectra (A) The SNPs of the putative *SM22.6 antigen* (A12) were checked by re-sequencing of PCR products amplified from genomic DNA samples of field-collected isolates of S. japonicum obtained from five Chinese provinces, as indicated at the right. The SNPs indicated by the red arrows were identical to the findings based on EST data in this study, and the SNP sites indicated by black arrows were not identified by us, according to the stringent criteria employed. Substitutions of amino acid residues due to the missense mutations are illustrated based on the DNA sequences. (B) An example for the verification of the translated peptide variants due to nonsynonymous SNPs by MS/MS spectra. The amino acid S (serine) of SJCHGC01743 protein was replaced here by a smaller amino acid G (glycine), where the b3 ion was shifted to the lower mass range as indicated by arrows. Both peptides were detected in the same protein extracts from female schistosomes by the mass spectrometry data. The position of the peptide within the protein sequence is highlighted with red color.

Some genes exhibited complex genetic variation (Figures 5A and S5). For example, putative *SM22.6 antigen* (A12) was selected for PCR-sequencing verification using genomic DNAs isolated from field samples of S. japonicum from five provinces of southern China: Jiangxi, Hunan, Hubei, Sichuan, and Anhui. The resulting sequences revealed that in addition to those found by the EST strategy, multiple SNPs in putative *SM22.6 antigen* (A12) were identified by the DNA sequencing (Figure 5A). This suggests that certain genes might include complex genetic variants involved in immune evasion and natural selection.

Indels (insertion/deletion length variations) represent another source of genetic polymorphism distinct from SNPs. There were 2,806 indel sites, including 2,225 in 948 clusters with CDSs, and 581 in 263 clusters without CDSs, apparent in 1,211 clusters with at least four ESTs. There were 1,078 (38.4%) indel sites in the coding regions of 560 genes with CDSs (Tables S5 and S6). The length of indels was primarily in the range of one to three nucleotides, with the majority, 2,459 (78.1%) of all 3,147 indels, exhibiting an insertion/deletion of just a single nucleotide. Significantly, some proteins deduced from the known genes with indels were localized on tegument or eggshell-containing samples. For example, the motor proteins (e.g., paramyosin), enzymes (e.g., peptidylprolyl isomerase), membrane proteins (e.g., 21.7 kDa antigen), and others (e.g., 14–3–3 epsilon and Hsp60) showed significantly higher indel frequencies than other genes, again suggesting that polymorphisms could confound effective host responses targeting these antigens. In addition, *legumain (antigen Sj32), phosphomannose isomerase* (type I), and *cytochrome c oxidase* (subunit 1) exhibited indel genetic polymorphisms.

Previous reports have demonstrated microsatellite polymorphisms in field and laboratory populations of S. mansoni [46] and S. japonicum [47]. In the present report, among a total of 14,962 consensus sequences, we identified 1,026 repeat motifs, in which there were 345 (33.1%) with at least ten di-nucleotide repeats, and 625 (60.1%), 70 (6.7%), and two (0.2%) with at least five repeats for tri-, tetra-, and penta-nucleotide repeats, respectively. The 444 microsatellite repeats were found in 411 clusters with CDSs of which 174 (39.1%) were localized in the protein-encoding regions, while 50 (11.2%) and 220 (49.5%) were found in 5′ and 3′ UTRs, respectively (Tables S5 and S6). This higher frequency of microsatellites localized within UTRs could contribute to the pronounced regulatory effects on protein translation and stabilization of mRNAs. The dinucleotide motif (TA)n was found commonly in 313 (91.5%) of 342 clusters with di-nucleotide repeats, whereas (CA)n and (GA)n repeats were found in 23 and nine clusters, respectively. Most of the microsatellite repeats (159 of 174, 91.4%) localized in protein-encoding regions were tri-nucleotide repeats, whereas these accounted for 146 (54.1%) of 270 microsatellites in UTRs, implying that some proteins might be prone to accumulate polymorphisms involving tri-nucleotide repeat microsatellites. Furthermore, some microsatellite repeats, including (TAA)n, (CAT)n, (CAT)n, (CAA)n, (TAG)n, (CAA)n, (TAA)n, (TGG)n, and (GAA)n were common within the protein-encoding regions (Figure 4C). The stretches of polymers of Asn (encoded by AAT), Asp (GAT), Ser (TCA), Gln (CAA), Thr (ACT, ACA), Ile (ATA), Pro (CCA), and Glu (GAA, encoded by specific microsatellite repeats) could have important molecular functions (Table S6). Moreover, 59 homopolymers with more than ten tandem amino acids were here found in 54 proteins, a low frequency as compared to that of Dictyostelium discoideum [48] and Plasmodium falciparum [49]. The stretches of polymers of Asn and Ser, as the most common homopolymers, occurred in 19 and 15 S. japonicum proteins, respectively, somewhat different from the situation in the genome of D. discoideum and *P. falciparum,* where poly N and Q or poly N and K are the predominant motifs, respectively [48,49]. Furthermore, I_33_ were the longest homopolymers encoded by microsatellite DNA repeats in the 8,420 S. japonicum CDSs (Table S6), where the DNA sequence composed of (TAA)33 tri-nucleotide repeats can encode the ATA/(I)33 homopolymer. Of 174 CDSs with microsatellite repeats, 52 gene products were identified by our proteomics analyses, including receptor kinase I-interacting protein (SIP), which appears to be localized on the tegument or eggshell at the host-parasite interface.

The proteomic data from the MS/MS spectra were further employed to identify the translated variants due to the nonsynonymous SNPs, indels, and microsatellites. Five peptide variants due to the nonsynonymous SNPs were identified by the MS/MS spectra, where both wild-type and a peptide variant of SJCHGC01743 protein were found to match perfectly with the proteomics data (Figure 5B and Table S6). This strongly suggests that the genetic polymorphisms by SNPs indeed may result in variant translated products.

### Conserved Proteins

**Figure 6:**
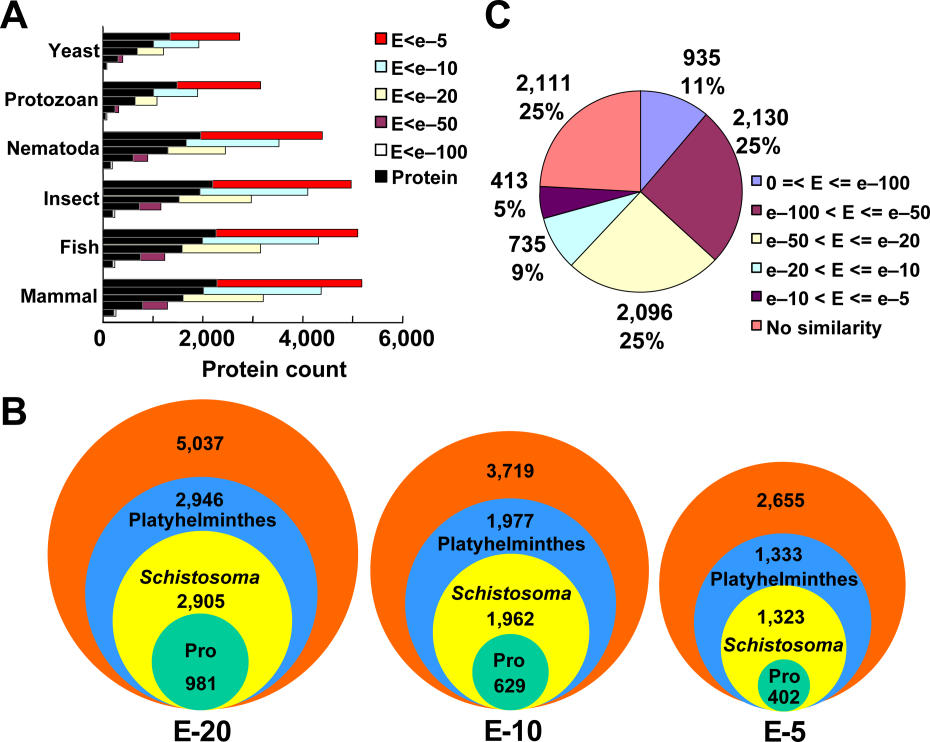
Comparative Genomic Analysis of S. japonicum Genes with CDSs from Representative Sequenced Genomes (A) The deduced S. japonicum proteins were compared with public protein datasets from mammalian hosts *(Homo sapiens, R. norvegicus,* and *M. musculus),* fish *(T. nigroviridis* and *T. rubripes),* insects *(A. gambiae* and *D. melanogaster),* nematodes *(C. briggsae* and *C. elegans),* protozoans (*P. yoelii nigeriensis* [17XNL], P. falciparum [3D7]), *(Cryptosporidium parvum* and *C. hominis),* and fungi *(S. cerevisiae)* at a cutoff of different BLASTP E values indicated by icons. The black bars indicated that the numbers of the deduced protein were identified by this proteomic analyses. (B) Potential Phylum Platyhelminthes- and *Schistosoma*-specific genes were predicted through interrogating the platyhelminth and *Schistosoma* ESTs in GenBank. The numbers within the largest (orange) circle indicate S. japonicum CDSs that were not similar to any known protein in a GenBank dataset, which excluded all flatworm sequences at a cutoff of different BLASTP E values. The numbers within the middle-sized blue and small yellow circles represent S. japonicum CDSs with similarity to the EST data from platyhelminths and schistosomes, respectively, at a cutoff of tBLASTN E value of 10^−20^. The numbers within the smallest green circles indicate proteins identified by the proteomic approach. (C) S. japonicum CDSs were compared with EST and cluster datasets of S. mansoni using the tBLASTN program at a cutoff of different E values indicated.

The 8,420 CDSs were further compared with the protein datasets derived from model organisms with sequenced genomes. Of translated CDSs, 38% or 62% were similar to mammalian proteins at BLASTP Expectation (E) values with less than 10^−20^ or 10^−5^, respectively (Figure 6A). Of these CDSs, 30%–37% showed significant similarity with proteins from fishes *(Tetraodon nigroviridis* and *Takifugu rubripes),* insects *(Drosophila melanogaster* and *Anopheles gambiae),* and nematodes *(Caenorhabditis elegans* and *Caenorhabditis briggsae),* at BLASTP E values of 10^−20^. Furthermore, only about 10% of the S. japonicum CDSs shared significant sequence similarity with proteins examined for the four Apicomplexan parasitic protozoa, P. falciparum (strain 3D7), *Plasmodium yoelii nigeriensis* (17XNL), *Cryptosporidium parvum,* and *Cryptosporidium hominis,* respectively*;* overall, a total of 1,092 (13%) S. japonicum CDSs were similar to those of these protozoan parasites. Less than 1,213 (14.4%) S. japonicum CDSs shared sequence similarity with yeast *(Saccharomyces cerevisiae)* proteins at an E cutoff value of 10^−20^ (Figure 6A).

To explore potential molecular mechanisms of the schistosome-mammalian host interplay, 1,336 CDSs with high similarity (E < 10^−50^) with mammalian genes were analyzed more extensively. Significantly, mammalian-like receptor and related proteins, including insulin receptor protein kinase RTK-2, receptor tyrosine phosphatase (gamma and delta), purinergic receptor P2X (ligand-gated ion channel, 4), dioxin receptor, vasopressin-activated calcium-mobilizing receptor, and feline leukemia virus (subtype-B) receptor were identified by the transcriptomic and proteomic approaches, implying that the parasite can accept certain hormone and cytokine signals from the mammalian host in addition to endogenous schistosome signals. However, functional evidence is needed to further support this hypothesis. Interestingly, among 820 (61.4%) of 1,336 highly conserved proteins identified with confidence by our proteomics approaches, 174 and 217 were potentially localized to the tegument and eggshell, respectively, i.e., at the host-parasite interface (Table S2).

These included numerous cytoskeleton and motor-associated proteins, chaperones, extra cellular matrix molecules, as well as enzymes involved in redox homeostasis, which could be involved with evasion of immune responses by antigenic mimicry, a strategy that has long been predicted (along with others including host antigen masking) to account for the chronic nature of schistosome infection [50]. Additionally, other immune-associated molecules including immunophilin, cyclophilin B, endoplasmin (gp96), and HLA-B associated transcript 1 may be contributing to the immune evasion and immune-dependent growth of the parasite by modulating the innate and adaptive immune systems of the mammalian host.

### Schistosome-Specific Proteins

Phylum Platyhelminthes- and genus *Schistosoma*-specific genes are potential targets for vaccines, drugs, and diagnostic reagents for schistosomiasis. We first analyzed all 8,420 potential CDSs by comparing them with the known genes of all other organisms except flatworms. This analysis revealed that 40%–68% of the S. japonicum CDSs had similarity with known genes at cutoff E values of 10^−20^ to 10^−5^, respectively, and indicated that the remaining clusters represented a resource for identifying potential candidate flatworm-specific and schistosome-specific genes. The remaining 32% −60% CDSs (at cutoff E values of 10^−20^ to 10^−5^) were then compared with the 223,321 public nucleotide entries deposited in GenBank for species belonging to the Phylum Platyhelminthes (except for S. japonicum), which included 12,621 entries for cestodes, 747 for monogeneans, 198,809 for digeneans, and 11,144 for turbellarians. The comparisons revealed that 50%–59% CDSs had significant similarity with the nucleotide entries at a cutoff of tBLASTN E value of 10^−20^, implying that these genes may be Phylum Platyhelminthes-specific (Figure 6B). Furthermore, to identify schistosome-specific genes, the candidate Platyhelminthes-specific CDSs with high similarity with nucleotide entries for the Phylum Platyhelminthes (tBLASTN < 10^−20^), were compared with 195,620 nucleotide entries only for the genus *Schistosoma* (except S. japonicum), including 195,414 from S. mansoni. These comparisons revealed that 50%–58% were similar to the available *Schistosoma* transcriptomic data at tBLASTN E value of 10^−20^ (Figure 6B). However, it should be pointed out that 61%–75% of all 8,420 S. japonicum CDSs were similar to the available S. mansoni transcriptomic data at tBLASTN, with expectation values of 10^−20^–10^−5^, respectively (Figure 6C). The S. japonicum genes share significantly closer identity with the genes of S. mansoni than with other organisms, which suggests that most schistosome genes share pair-wise orthologs between S. japonicum and *S. mansoni.* Therefore, 1,323 CDSs representing stringent genus *Schistosoma*-specific genes warrant consideration as candidate targets for new interventions. Moreover, 402 (30.1%) of 1,323 gene products were confidently identified by our proteomics approaches, where 111 and 102 were found in cercariae and hepatic schistosomula, respectively; while 26 and 50 proteins, including Sj-Ts4 and MF3 appeared to be tegument and eggshell proteins, respectively (Table S2).

## Discussion

Using a transcriptomics approach, it has been estimated that S. mansoni has a complement of ~ 14,000 genes [8]. The 14,962 gene clusters, generated from a new suite of 84,449 high-quality ESTs from egg, larval, and adult developmental stages of S. japonicum appeared to represent 8,420 potential CDS-encoding proteins, which accordingly probably represent 60%–70% of all S. japonicum proteins (if we assume that the gene number of S. japonicum and S. mansoni is about the same). Moreover, 3,260 proteins, accounting for 38.7% of 8,420 potential CDSs, were confidently identified throughout the parasite life cycle using high-throughput proteomics approaches, implying that the *S. japonicum* transcriptomic dataset is a relatively reliable resource of genetic information. Notably, these 3,260 proteins identified by our proteomics approaches represent a more than 100-fold increase in the number of schistosome proteins so far reported using similar approaches [51,52]. Furthermore, by describing the presence of numerous SNPs and indels in many S. japonicum genes, we have revealed extensive genetic polymorphism in this parasite, which should resolve the long-standing debate over the extent of genetic heterogeneity in populations of the Oriental schistosome [53,54].

Most genes of S. japonicum and S. mansoni appear to share pair-wise orthologs because 5,161 (61.3%) of the new 8,420 S. japonicum gene sets were similar to the S. mansoni EST data with limited transcriptomic information at a tBLASTN E value of 10^−20^. It can be expected, as more sequence data become available, that additional comparative genomic analysis between both these two species will provide more pair-wise orthologs, including single-copy genes and gene families with many paralogs. Phylum Platyhelminthes- and *Schistosoma*-specific genes can be considered to be potential candidates for new drugs and vaccines, in like fashion to the situation with parasites from the phylum Nematoda [55]. Our comparative genomic analysis revealed that at least half of the CDSs, without similarity to known genes from all organisms other than platyhelminths, could be considered as *Schistosoma* genus-conserved genes across the genus due to orthologs known from the *S. mansoni* transcriptome.

The highly co-evolved relationship of schistosomes and their hosts appears to include exploitation of host endocrine and immune signals, although the molecular mechanisms involved in the host-parasite interplay remain poorly understood [2–4]. Characterization of these key genes and their cognate proteins related to the parasite-host interplay should lead to a better understanding of this intriguing biological phenomenon. Together with the potential for accepting the mammalian-derived hormones, cytokines, chemokines, and immune cells that facilitate parasite growth, development, and maturation, various schistosome motor proteins and chaperones may play key roles in avoidance of immunological attack and maintenance of parasitism and parasite survival through antigen mimicry strategies. The presence of other protein groups at the interface, including anti-oxidant enzymes and protease inhibitors, supports the notion that they play roles in facilitating parasite evasion of host immunological responses [56,57]. In addition, immunity-related molecules with strong similarity to host proteins, including immunophilin, cyclophilin B, endoplasmin (gp96), and HLA-B associated transcript 1 were localized on the schistosome tegument or eggshell. These proteins can be expected to contribute to the immunopathology and chronicity associated with schistosomiasis, by facilitating escape from host immunosurveillance mechanisms through molecular mimicry, antigen presentation, and immune modification or immune inhibition.

A recent, timely reassessment of schistosomiasis-related disability [58], combined with new information on the global prevalence of schistosome infection [1], indicates that the true public health burden of schistosomiasis is substantially greater than previously appreciated. The abundance of new gene and protein sequences of S. japonicum reported here should lead to a more fundamental understanding of the biology of this important human parasite and the molecular mechanisms underpinning the pathology of schistosomiasis. Furthermore, we anticipate that this new information will contribute significantly to the elucidation of complete sequences for the schistosome genome, proteome, and transcriptome, which in turn can be expected to provide new insights for the development of novel interventions leading to improved treatment and control of schistosomiasis.

## Materials and Methods

### Schistosome materials.

A field-collected isolate of S. japonicum from Anhui Province, China was used in all of the transcriptome and proteome investigations. To evaluate whether genetic polymorphisms occur in natural, geographically discrete populations of *S. japonicum,* additional Chinese field isolates were collected from Jiangxi, Hunan, Hubei, and Sichuan Provinces. Cercariae of S. japonicum were shed from naturally infected *Oncomelania hupensis hupensis* snails collected in the field from these provinces. In addition, *O. hupensis hupensis* were infected in the laboratory with miracidia hatched from eggs. Each rabbit and mouse was experimentally infected percutaneously with 1,000 and 100 cercariae, respectively. Developing unpaired hepatic schistosomula were isolated from livers of experimentally infected mice at 14 d post-cercarial challenge, and adult worms and eggs were obtained from the mesenteric veins and liver of infected mice or rabbits, respectively, at 42–45 d post-infection. Adult parasites were manually separated into male and female worms with the aid of a microscope. Eggs, miracidia, cercariae, hepatic schistosomula, male, female, and mixed-sex adults were washed thoroughly in PBS to remove host cell debris, and then stored at −80 °C for up to 6 mo. To obtain eggshells after miracidia had been freshly hatched, eggs collected from infected livers were incubated in distilled water under a bright light for 6 h at room temperature [59]. After the miracidia were removed, the remaining eggshell-containing pellet was collected by centrifugation, and after washing three times with PBS, examined by light microscopy to ensure it contained empty eggshells only. Schistosome tegumental preparations were isolated from hepatic schistosomula, mixed-sex, and separated male and female adult worms for the proteomic survey using an optimized Triton X-100 detergent-based technique [25].

### cDNA libraries and DNA sequencing.

We isolated poly (A)^+^ mRNA from total RNA on oligo-dT sepharose (Qiagen, Valencia, California, United States), after extracting total RNA from the frozen schistosome life-cycle stages using TRIzol Reagent (GIBCO-BRL, San Diego, California, United States). We employed the poly (A)^+^ RNAs from hepatic schistosomula, mixed-sexed adults, and miracidia to construct new cDNA libraries with long inserts (larger than 2 kb) in the directional phage vector Uni-ZAP XR, using oligo dT priming (Stratagene, La Jolla, California, United States). Long-read DNA sequencing was carried out on an ABI 3730 DNA sequencer (ABI, Columbia, Maryland, United States) on clones selected randomly from the new cDNA libraries and the representative clones derived from the assembled clusters with potential CDSs. All EST sequences were quality-trimmed through Phred 20 prior to assembling the data. Phred was employed as a base-calling program to evaluate the quality of raw EST sequences by assigning an error probability to each base. A Phred score of 20 for a given peak in the sequence chromatogram indicates that a base is incorrectly called one time in every 100 bases, and so, in general, a Phred 20 means that the sequence is reliable.

### cDNA assembly and ORF prediction.

The cDNA assembly procedure and prediction and annotation of CDSs were carried out according to the stringent criteria described in the Protocol S1. Analysis of statistical significance of gene expression was performed using tools available at http://www.igs.cnrs-mrs.fr/~audic/significance.html [12].

### Comparative genomic analysis.

Comparative genomic analysis was performed using BLAST programs based on public nucleotide and protein databases via the public access Web sites: GenBank at National Center for Biotechnology Information (NCBI) (http://www.ncbi.nlm.nih.gov) for gene, protein, and EST entries; Ensembl (http://www.ensembl.org) for human, *Rattus norvegicus, Mus musculus, T. rubripes, A. gambiae, D. melanogaster, C. briggsae,* and *C. elegans;* http://www.genoscope.cns.fr/externe/tetraodon for *T. nigroviridis;* http://www.plasmodb.org for *P. yoelii nigeriensis* (17XNL) and P. falciparum (3D7); http://www.hominis.mic.vcu.edu for *C. hominis;* and GenBank and http://bioinfo.iq.usp.br/schisto for S. mansoni genes and ESTs. We also prepared a local copy of the S. mansoni dataset on our Web server under the link http://function.chgc.sh.cn/sj-proteome/download/download.php. Conserved protein domains or motifs and families were identified by interrogation of the InterPro protein domain database version 7.0 (http://www.ebi.ac.uk/interpro) using S. japonicum protein sequences deduced from putative CDSs as queries. The CDSs that were similar to known genes and domains were further assigned into different molecular functions and biological processes based on GO (http://www.geneontology.org). Signal peptide and transmembrane predictions were accomplished using on-line tools at http://www.cbs.dtu.dk/services/SignalP and http://www.cbs.dtu.dk/services/ TMHMM. The subcellular localization of CDSs was predicted using PSORT II at http://www.psort.nibb.ac.jp/form2.html.

### Genetic polymorphisms.

Candidate SNPs or indels were identified based on high-quality assembled contigs with multiple ESTs using revised stringent criteria [44,45] as detailed in Protocol S1.

### The ratio of dN/dS.

Orthologous gene pairs between S. japonicum and S. mansoni were identified as reciprocal best BLAST (BLASTP version 2.2.12) hits using translated protein sequences. Alignments with greater than 50% similarity in length and with E < 10^−10^ were considered significant. The 1,514 orthologous gene pairs from S. japonicum and S. mansoni were globally aligned with ClustalW version 1.83 (default parameters). The dN and dS between pair-wise alignments were calculated with SNAP (http://www.hiv.lanl.gov/content/hiv-db/SNAP/WEBSNAP/SNAP.html) [60] based on the method of Nei and Gojobori [61].

### 2D-nano-LC-MS.

The protein mixtures from solubilized schistosome samples were digested and then fractionated into 20–30 subgroups by strong cation exchange (SCX) chromatography. The peptide mixture of each SCX fraction was sequentially loaded onto a reverse phase (RP) trap column, connected in-line to a C18 column (LC Packings Incorporated, San Francisco, California, United States), as published [10], and the peptide mixture was eluted into a QSTAR pulser i mass spectrometer coupled to a Protana NanoES electrospray ionization source. The remaining supernatant and the insoluble pellet were denatured in the loading buffer for SDS-PAGE and size-fractionated by one-dimensional electrophoresis. Each lane of the gel was cut equally into eight or 12 slices that were subsequently in-gel digested with trypsin. Silver staining and the in-gel tryptic digests were performed according to standard procedures [62]. Hydrolysates from gels were likewise analyzed by in-line RP-LC-MS as described above (further details in Protocol S1).

### MS data interpretation.

The MS/MS spectra were searched against the rabbit or mouse and the S. japonicum protein databases, the latter deduced from CDSs obtained from ESTs in the present study, using MASCOT software (http://www.matrixscience.com, Matrix Science). The initial results were combined and further filtered using revised criteria [10]. Protein quantifications were carried out as previously described [11] (more detailed in Protocol S1). The proteomic data were further employed to identify the translated variants due to the nonsynonymous SNPs, indels, or microsatellites, where a special peptide database containing the protein variants through/beyond the genetic polymorphisms was established. The MS/MS spectra were searched against both the specific and common databases by MASCOT software. The matched peptide variants with MASCOT score higher than 30 were considered as significant, as the possibility that the MS/MS spectra were matched to other common peptides was excluded.

### Immunofluorescence assay.

Immunofluorescence assays were carried out on 5-μm-thick frozen sections of adult worms embedded in OCT fixative. Slides were incubated in a humid atmosphere at 37 °C for 60 min with an anti-immunophilin (SjFKBP50) monoclonal mouse antibody, generated by immunizing mice with recombinant SjFKBP50 protein. The slides were washed and incubated for 60 min with a FITC-conjugated, rabbit anti-mouse immunoglobulin antibody (Nordic, Tilburg, Netherlands), diluted 1/40 in PBS containing 0.5 mg/ml Evans blue in a humid atmosphere at 37 °C. Antibody staining was visualized and recorded using a Leica DM-RB fluorescence microscope (Leica, Wetzlar, Germany) with the appropriate filter combination for FITC fluorescence.

## Supporting Information

Figure S1 Schematic Representation of the Strategy for the Integrated S. japonicum Transcriptome and Proteome Analyses The boxes represent the main results or steps of the transcriptomic or proteomic analyses that were described in the main text. The arrows illustrate the direction of process procedures.
(8 KB PDF)

Figure S2 Characteristics of the S. japonicum Clusters (A) The length distribution of all clusters, clusters with protein-coding genes, and with complete CDSs of *S. japonicum.* nt, nucleotide genes.
(B) The length distribution of proteins deduced from all CDSs and complete CDSs of *S. japonicum.* aa, amino acid.
(5 KB PDF)

Figure S3 Predicted Functions of S. japonicum CDSs and Proteins Based on GO (A) The distribution of GO categories based on molecular functions (left) and biological processes (right). The filled and open boxes represent the GO categories according to the transcriptomic and proteomic data, respectively. The classification integrated the numbers of known genes and InterPro domains assigned into different GO categories.
(B) The distribution of GO categories throughout the life cycle, including C, cercariae; S, hepatic schistosomula; A, adults; E, eggs; and Mi, miracidia based on the proteomic data, according to biological processes (left) and molecular functions (right). The detailed list of GO assignments can be found in Tables S2 and S4.
(12 KB PDF)

Figure S4 Correlation between Transcriptomic and Proteomic Data of Representative Proteins among Life-Cycle Stages and Sexes (A) Some developmental stage-enriched proteins were generally consistent with the transcriptomic data. C, cercariae; S, hepatic schistosomula; A, adults; E, eggs; and Mi, miracidia.
(B**)** Gender-enriched proteins are also shown as colored boxes: F, females; M, males. The protein abundances were calculated as detailed in the Protocol S1. Proteins not detected in life-cycle stages are depicted as black blocks. The abundances of mRNAs are represented by the ratio of EST copy numbers to the total EST numbers in the libraries.
(602 KB PDF)

Figure S5 Verification of SNPs by DNA Sequencing (A) *Fructose 1,6 bisphosphate aldolase.*
(B) Putative *preprocathepsin L.*
(C) *SJCHGC00098,* containing similarity to *preprocathepsin cathepsin L,* was checked by re-sequencing PCR-amplified products of field S. japonicum genomic DNA samples from five Chinese provinces, indicated on the right. The SNPs indicated by the red arrows were identical to the EST findings presented in this study. The single letter amino acid codes separated by a forward slash represent homozygous or heterogeneous SNPs in the genome, and the numbers indicate the position of the SNP sites on the cluster sequences. Replacements of amino acid residues due to missense mutations are illustrated based on the DNA sequences.
(621 KB PDF)

Protocol S1 The Detailed Approaches for Transcriptomic and Proteomic Analyses (46 KB PDF)

Table S1 Summary of S. japonicum Transcriptomic Data (17 KB XLS)

Table S2 Integrated Information of the Transcriptome and Proteome of S. japonicum
(7.4 MB XLS)

Table S3 Domain Analyses of S. japonicum Proteins (463 KB XLS)

Table S4 GO Classification of S. japonicum Transcripts Based on Similarity with Known Genes and Domains (30 KB XLS)

Table S5 Statistics of Polymorphisms of S. japonicum Clusters (18 KB XLS)

Table S6 Polymorphisms of S. japonicum Clusters (1.3 MB XLS)

### Accession Numbers

The nucleotide sequence described here has been deposited in public databases with accession numbers: EST sequences (CV671092–CV674724, CV581651–CV582043, CV693277–CV699272, CV681278–CV693276, CV736204–CV758494, and CX856533–CX863389); the full-length, partial cDNAs and hypothetical genes (AY812752–AY816180, AY808309–AY812729, and AY914876–AY915917).

The GenBank (http://www.ncbi.nlm.nih.gov/Genbank) accession numbers for *schistosoma japonicum* sequences described in this paper are *acetylcholine receptor (alpha-3 chain)* (AY815304), *actins* (AY813805), *amidase* (AY809279), *21.7 kDa antigen* (AAD13338), *budding uninhibited by benzimidazoles 1* (AY812514), *calcineurin A* (AY810505), *calcium/calmodulin-dependent protein kinase II (delta isoform 3)* (AY813551), *calcium-binding protein Sj66* (AAC62193), *calpain* (AY808568), *calreticulin* (AAC00515), *collagen (type I alpha 3)* (AY810097), *craniofacial development protein 1* (AY814915), *cyclophilin B* (AY816130), *cytochrome c oxidase (subunit 1)* (AAG13143), *dioxin receptor* (AY813606), *dynein light chain 1* (AAD41626), *eggshell protein* (AAP05897 ), *egumental antigen Sm20* (AY813791), *endoplasmin (gp96)* (AY813390), *14–3–3 epsilon* (AY815015), *extracellular superoxide dismutase* (AY812195), *F-box only protein 9* (AY815855), *feline leukemia virus (subtype-B) receptor* (AY813727), *female-specific 800 protein* (AY815492), *fimbrin* (AY809033), *flavoprotein (Fp)* (AY814217), *forebrain embryonic zinc-finger like (Fezl)* (AY808322), *G protein alpha subunit* (AY815795), *GABA receptor* (AY815726), *glutathione-S-transferase* (AY816103), *gynecophoral canal protein* (AY810721), *high voltage-activated calcium channel (beta subunit 2)* (AY812476), *histone H2A (gonadal)* (AY812081), *HLA-B associated transcript 1* (AAP06453), *Hsp60* (AY813151), *immunophilin (FK506-binding protein 50)* (AY815389), *insulin receptor protein kinase RTK-2* (AY813034), *karyopherin* (AY810727), *leucine aminopeptidase* (AY814468), *manganese superoxide dismutase* (AY814748), *MF3* (AY809998), *microtubule-associated protein* (AY812854), *myosin* (AY810340), *nitric oxide synthase 1 (NOS1)* (AY815837), *nocturnin* (AY812870), Notch receptor (AY810632), *osteonectin (SPARC)* (AY814549), *p40 major egg antigen* (AY813596), *p48 eggshell protein* (AY812971), *paramyosin* (AAD29285), *peptidylprolyl isomerase* (AY814078), *phosphomannose isomerase (type I)* (AY812397), *platelet glycoprotein IIIa (GPIIIa)* (AY810920), *presenilin* (AY809924), *prion protein interacting protein* (AY815835), *prosaposin* (AY815893), *protein disulfide isomerase* (AAC78302), *purinergic receptor P2X* (AY812469), *putative C1-tetrahydrofolate synthase* (AAP06003), *Putative deoxyribodipyrimidine photo-lyase (DNA photolyase)* (AY812553), *Putative ribophorin II* (AY809963), *putative ribosome-associated protein P40* (AAP05908), *putative sex-determining region Y protein (SRY)* (AY813503), *receptor tyrosine phosphatase (gamma and delta)* (AY812724), *regulator of G-protein signaling 2* (AY814274), *SJCHGC01743 protein* (AY813753), *Sj-Ts4* (AY812897), *SM22.6 antigens (A12)* (AY813797), *SnaK* (AY808337), *stage-specific protein SPO-1* (AY812887), *Su(var)3–9* (AY815180), *22.6 kDa tegument membrane-associated antigen* (AY815413), *thioredoxin peroxidases* (AY813893), *transducin-like enhancer of split 3 (TLE3)* (AY810007), *trispanning orphan receptor* (AY814912), *tropomyosin* (AY809967), *tubulins* (AY815746), *twister* (AY809513), *ubiquitin-protein ligase NEDD4-like* (AY812719), and *vasopressin-activated calcium-mobilizing receptor (cullin 5)* (AY812566). The Swissprot (http://www.ebi.ac.uk/swissprot) accession number for the *schistosoma japonicum* sequence described in this paper is antigen Sj32 (P42665). The PIR (http://pir.georgetown.edu) accession number for the *schistosoma japonicum* sequence described in this paper is cathepsin B-like cysteine protease precursor (pir||S31909).

All transcriptomic and protemic data described here are freely available and can be downloaded from our Web site: http://www.function.chgc.sh.cn/sj-proteome/index.htm.

## Abbreviations

2D-nano-LC: two dimensional-nano-liquid chromatography
bp: basepair
CDS: coding sequence
dN: nonsynonymous substitution
dS: synonymous substitution
EST: expressed sequence tag
indel: insertion/deletion length variation
GC: guanine-sytosine
GO: Gene Ontology
MS/MS: tandem mass spectrometry
SNP: single nucleotide poymorphism
