# Supplementary material for: New Perspectives on Host-Parasite Interplay by Comparative Transcriptomic and Proteomic Analyses of Schistosoma japonicum
Source: PLoS Pathog. 2006 Apr 14;2(4):e29. doi: 10.1371/journal.ppat.0020029 (PMC1435792; doi:10.1371/journal.ppat.0020029)
Supplement: Supplementary file 1 [file ppat.0020029.sd001.pdf]

Protocol S1:

Tegument isolation. The teguments were isolated from male, female, mixed-sex adult and hepatic schistosomula for proteomic surveys using an optimized Triton X-100 technique [1]. Briefly, the worms were rinsed three times with Krebs'-Ringer's, Tris-maleate-buffered saline (KRTM) [2] before suspension in KRTM/0.2% Triton X-100 solution. The mixtures were incubated in an icebath-shaker for 10 min with agitation. Subsequently, the tubes containing worms and fluid were vortexed mixed briefly and then placed in an ice bath to let the worms settle to the bottom. The supernatant fluid was removed with a pipette and the crude tegument pelleted by centrifuging the fluid at 2,500 × g for 15min. Both membrane isolates and worm carcasses were then analyzed by mass spectrometry approaches.

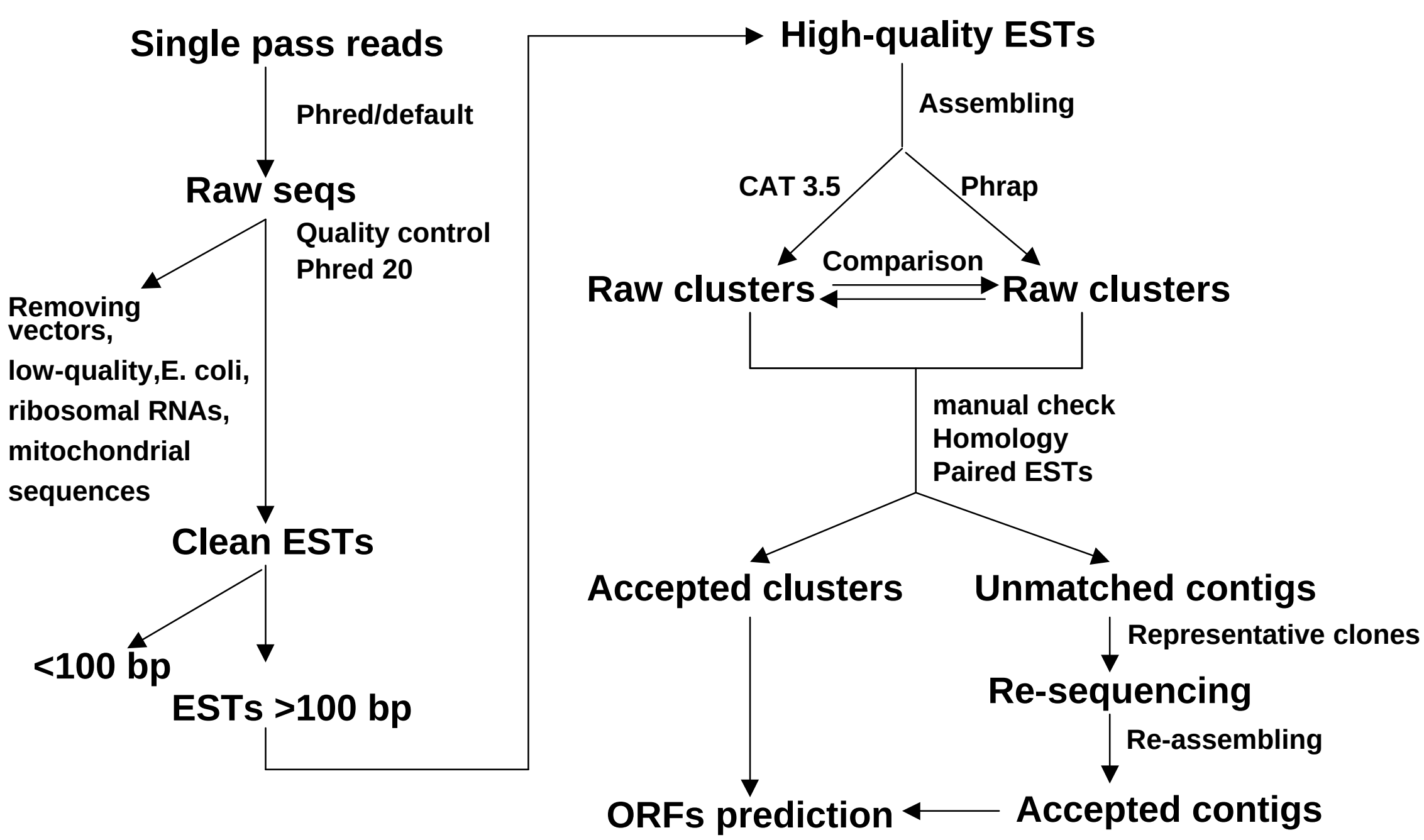

Figure 1. EST assembly procedure.

cDNA assembly. Repetitive, ambiguous and vector sequences were removed and ESTs shorter than 100 bp were excluded from the analysis, even if high-quality sequence. Then, the remaining ESTs, with a length greater than 100 bp, were clustered into longer contigs using Phrap (version 0.990329, http://www.phrap.org/) and CAT 3.5 (Clustering & Alignment Tools, Doubletwist, www.doubletwist.com) using the steps shown in Figure 1 of the Supporting Methods. We bridged contigs predicted to belong to the same genes, taking the advantage of paired end-reads from the same clones. Moreover, we re-sequenced representative clones from clusters with potential coding sequences (CDSs) lacking 5' or 3' ends. Generally this extended the contigs or provided connections with other clusters by long-read sequencing through a reassembling process. In this process, CDSs were iteratively predicted and revised for obtaining intact CDSs when the chosen ESTs were further reverse-sequenced. Finally, cluster sequences were manually checked and validated using Autoassembler 1.4 and DNA strider 1.2 (PERKIN ELMER) if ambiguity existed. The assembly procedures are illustrated in Figure 1 of the Supporting Methods.

CDS prediction and annotation. CDS prediction and annotation were carried out according to the stringent criteria as depicted in Figure 2 of the Supporting Methods. Briefly, CDSs were predicted according to the similarity (similar to known genes at a BLASTP cutoff of expectation (E) value of 10^{-10} or known domains), protein identification (>420,000 MS/MS spectra matching with 3,260 putative proteins), or length (hypothetical genes encoding at least 100 amino acids) (See Supplementary Fig. 1). Finally, 8,420 clusters were considered as having potential CDSs. Complete CDSs were predicted according to their orthologs in other organisms (identity of amino acid

sequences with more than 50% of the entire protein length and BLASTP cutoff of E value 10^{-20}), the typical Kozak sequences around potential first ATG, and the upstream in-frame stop codon of the putative first ATG respectively, in addition to a poly(A) stretch at the 3'-end. The in-frame stop codon located in the upstream of the putative first ATG would indicate that the CDS could not be extend by its 5' terminus and the downstream ATG was surely the first ATG of the CDS. The sequences missing either the first ATG or the terminating stop codon (partial CDSs) were annotated as partial ORFs if they were similar to the proteins of other organisms (BLASTP cutoff of E value 10^{-20}). The remaining CDSs were considered as hypothetical genes if they had weak similarity to known genes, domains and motifs, or if they encoded more than 100 amino acid residues. The annotation of these CDSs was performed according to similarity with known genes and proteins deposited in public databases, where the CDSs were directly nominated as names of orthologs or similar to ~, as illustrated in Figure 2 of the Supporting Methods. Hypothetical genes containing InterPro protein domains or motifs are also described in the annotation.

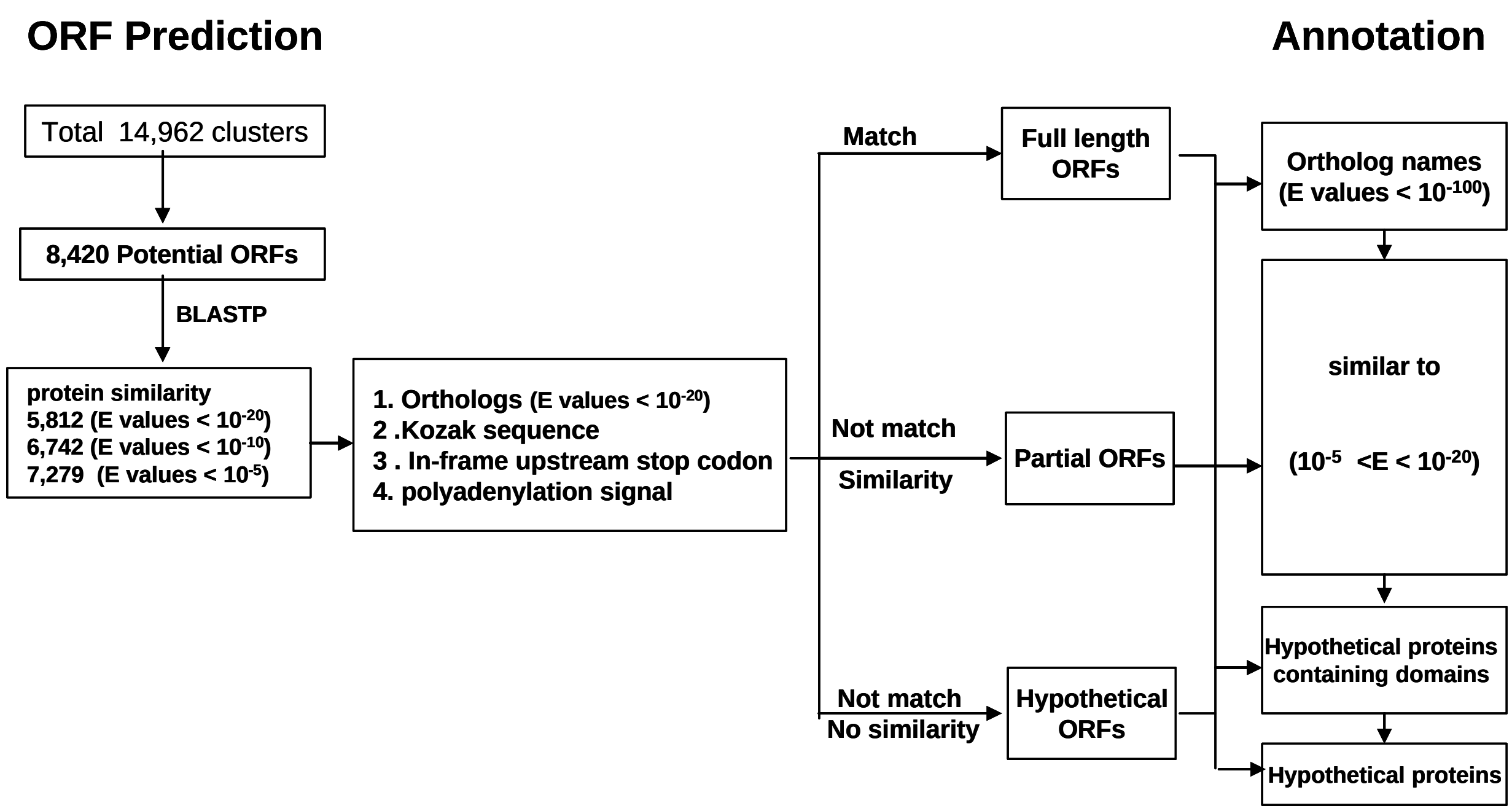

Figure 2. CDS prediction and cluster annotation.

Genetic polymorphisms. Candidate SNPs or Indels were identified based on high-quality assembled contigs with at least four ESTs [3,4], as depicted in Figure 3 of the Supporting Methods. (1) The contigs with candidate SNPs or Indels have a redundancy of four or more ESTs from two or more cDNA libraries, and each polymorphic nucleotide site was sustained by at least two ESTs; (2) Phred scores at the polymorphic sites were larger than 30; (3) the flanking 10 nt around the polymorphic sites have Phred scores larger than 20. In addition, at least ten repeats for di-nucleotide and at least five repeats for tri-, tetra-, and penta-nucleotide were here considered as the criteria to establish the presence of microsatellite motifs. To further evaluate candidate SNPs, PCR amplification

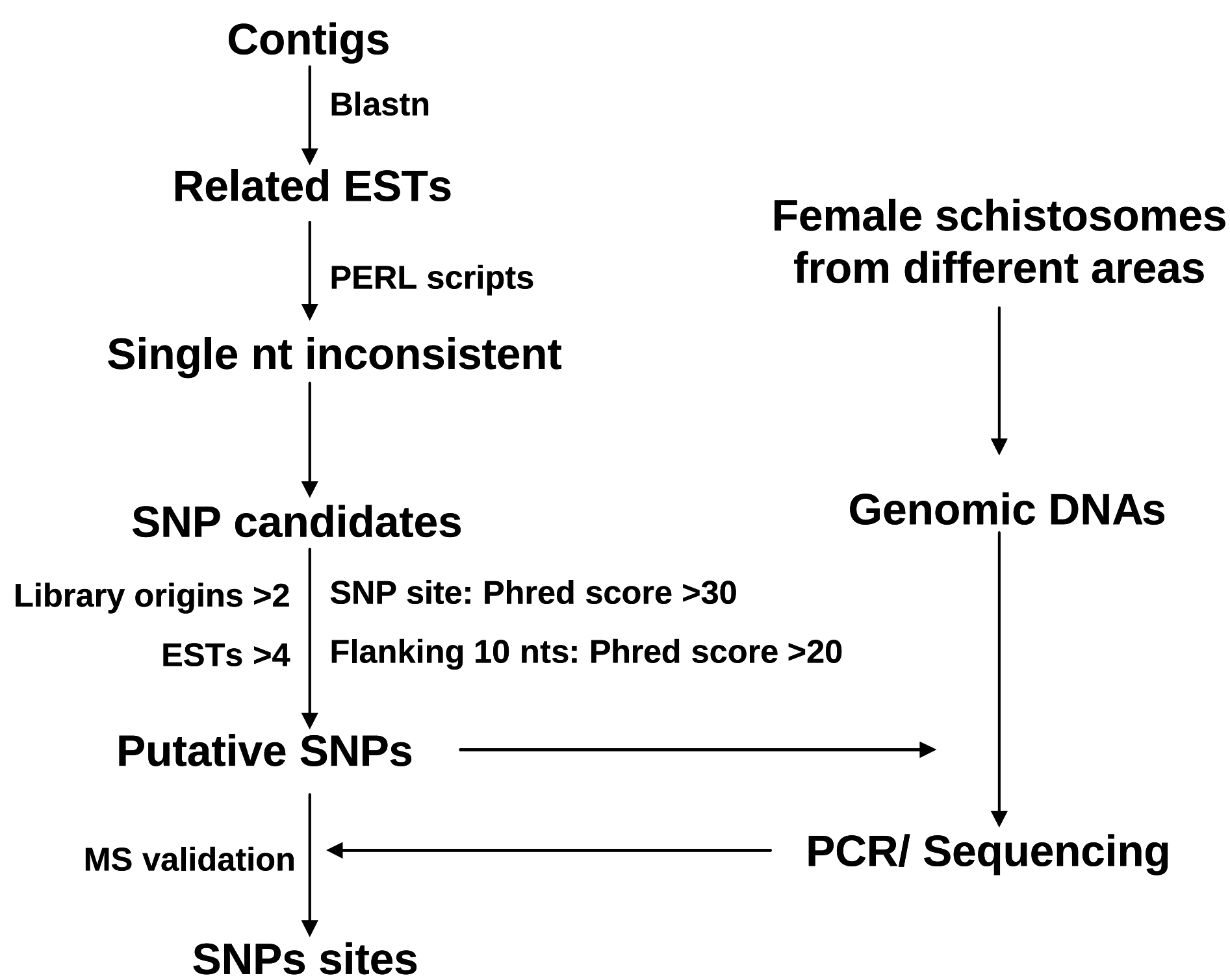

Figure 3. SNP analysis strages

was performed using genomic DNA samples extracted from field isolates of separated female adult *S. japonicum* worms, (the karyotypes of which include all the euchromosomes and sex chromosomes (ZW type) since females are the heterogametic

gender of schistosomes), collected from Jiangxi, Hunan, Hubei, Sichuan and Anhui provinces. PCR amplified products were sequenced using an ABI3700 sequencer and sequences quality-trimmed using Phred 20.

Protein samples and two-dimensional-nano-liquid chromatography-mass spectrometry (2D-nano-LC-MS). Schistosome samples, including cercariae, hepatic schistosomula (intact worms, teguments), adults (male, female and mixed sexes adults, and teguments), miracidia, and eggs (eggs and egg shells), were lysed by sonication in the presence of a cocktail of protease inhibitors (Sigma, P8340), respectively. The lysate was further solubilized by addition of sodium dodecyl sulfate (SDS) to 0.5%. After the lysate was centrifuged, the protein mixture (500-1,000 μg) from the supernatant was treated with 10 mM dithiothreitol (DTT), and then carboxyamidomethylated in 55 mM iodoacetamide. Next, the protein mixture was diluted with deionized water and then digested with modified bovine trypsin (Roche). The concentration of SDS was lower than 0.1% in the reaction mixture. The resulting peptide hydrolysate was fractionated into 20-30 subgroups by strong cation exchange (SCX) chromatography using a 4.0 mm × 150 mm POROS 50 HS column (Sciex-Applied Biosystems). The volume of each SCX fraction was adjusted to ~30 μl using a vacuum lyophilizer. After this, the peptide mixture of each SCX fraction was loaded onto a reverse phase (RP) trap column (C18, 5 μm, 300 Å, 300 μm i.d × 5 mm, LC Packings) for desalting, at a flow rate of 10 μl/min. The trap column was sequentially connected in-line with a analytical 75 μm × 150 mm C18 column (LC Packings) and the peptide mixture was eluted into a QSTAR pulser i mass spectrometer coupled to a Protana NanoES electrospray ionization source at a flow rate of 200 nl/min. The Agilent 1100 capillary LC system (Agilent Technologies) was used to

deliver mobile phases A (0.5% acetic acid in water) and B (0.5% acetic acid in 100% acetonitrile) at a linear gradient from 5% B to 50% B within 60 minutes, along with a gradient from 50% B to 90% B within 30 minutes and a hold at 90% B for 15 minutes. A spray voltage of 2,500 was applied to a 10-µm i.d PicoTip nanospray emitter (New Objective) connected at the end of the analytical column through a stainless steel joint union (Valco Instrument) for a steady spray. The MS/MS spectra were recorded with information-dependent acquisition (IDA) and duty-cycle enhancement. The nine most intense ions with 2 to 4 charges in each survey scan were fragmented with rolling collision energy. The remaining supernatant and the insoluble pellet were denatured in the loading buffer for SDS-polyacrylamide gel electrophoresis (SDS-PAGE) and size

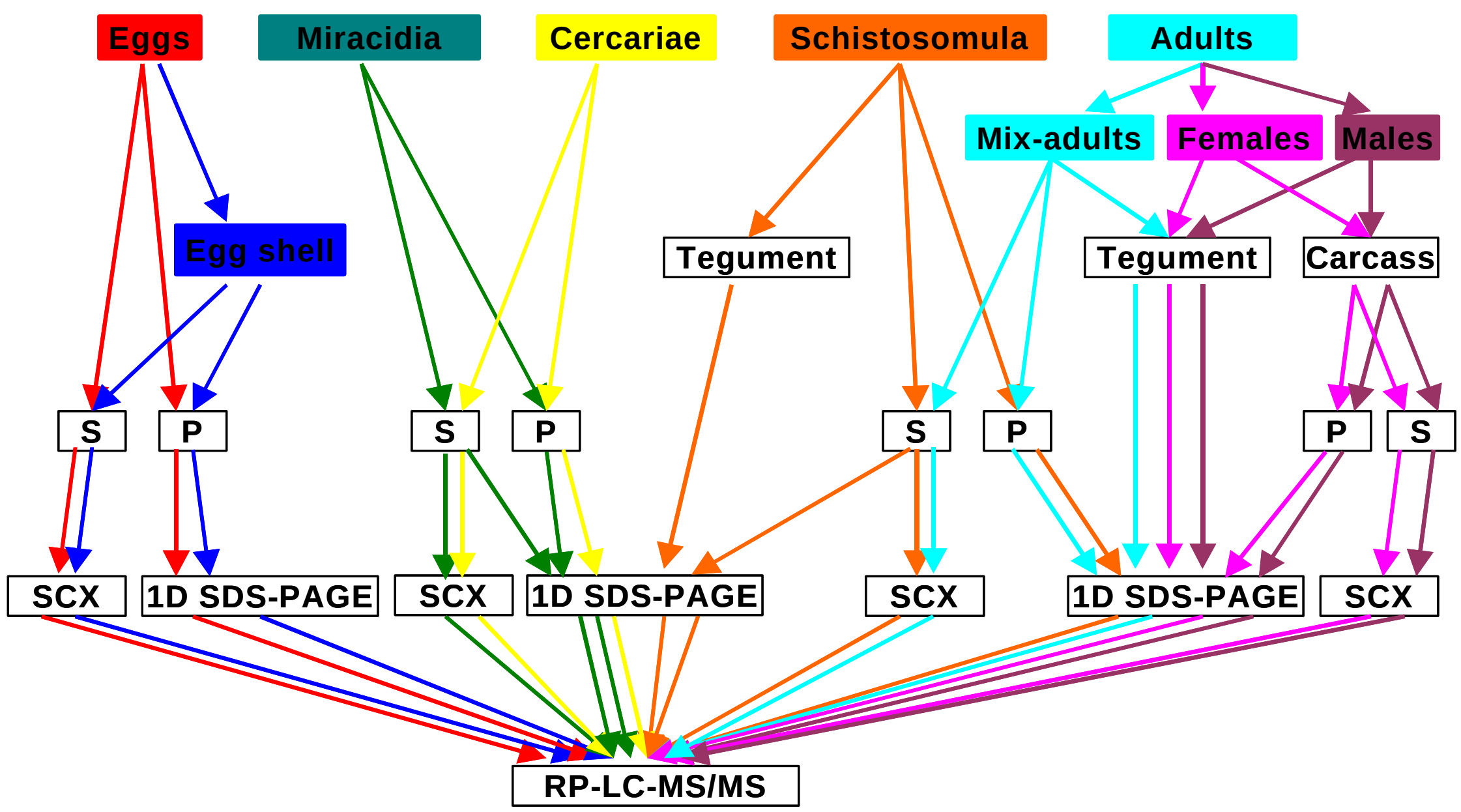

Figure 4. Proteomic analysis workflow

Note: P: pellet of the cell lysates; S, supernatant of the cell lysates.

fractionated by one-dimensional electrophoresis. Each lane of the gel was equally cut into eight or twelve slices, and the proteins within these slices were treated with DTT and iodoacetamide and in-gel digested with trypsin. The silver staining and the in-gel tryptic

digestion were performed according to an optimized procedure [5]. Afterwards, peptide mixtures from the gel were analyzed by on-line RP-LC-MS as detailed above. More than 400 nano-LC-MS/MS were performed and more than 420,000 tandem mass spectra were recorded. The strategy for the *S. japonicum* proteomics work is illustrated in Figure 4 of the Supporting Methods.

Mass data interpretation. All mass spectra were calibrated iteratively using known auto-cleaved peptides of bovine trypsin or other high score peptides revealed by initial database searching. A *S. japonicum* protein and peptide database was assembled using datasets deduced from the transcriptomic analyses. The redundant protein sequences were removed from the database if their sequences shared 95% identity with other sequences within 90% protein length. To remove contaminating proteins of host and other origins, a combined database including translations of all rabbit sequences in GenBank, an IPI mouse protein dataset containing 41,916 sequences, as well as bovine trypsin and human keratins, were used to filter MS/MS spectra. We used MASCOT software (http://www.matrixscience.com/, Matrix Science) for peptide assignment. The parameters included three maximum missed cleavages, monoisotopic, peptide charges +1/+2/+3, carbamoylmethylation for cysteine and oxidation for methionine. The information, such as peptide assignments, score, modification, delta mass, missed cleavage sites and peptide mono masses, were extracted from the raw MASCOT searching results using Perl scripts. Then, these primary datasets from different SCX or gel fractions of one sample were combined and further filtered automatically with Perl scripts according to specific criteria as follows. (1) For a single MS/MS spectra search, the top rank peptide assignments was considered, whereas the lower ranked peptide assignments were

discarded without further consideration; (2) candidate peptides without internal KR, KK, RK, or RR were accepted [6]; (3) peptides with more than 8 amino acids were accepted; (4) if a protein contained redundant peptides with different MASCOT scores, we only summed up the highest score of these redundant peptides when calculating the combined protein score; (5) proteins with combined MASCOT scores of higher than 60, after removing unacceptable peptides, according the first three criteria, were considered as significant identification; and protein hits with lower scores (26-60) were manually verified or rejected. Peptides matching with proteins from hosts and others were considered to be potentially contaminating proteins and these were discarded from the search. The molecular masses and pIs were calculated using Bioanalyst 1.1 (Applied Systems & MDS Sciex). The average sequence coverage was 27.4% (Cercariae, 25.5%; hepatic Schistosomula, 42.4%; Females, 17.8%; Mixed sex adults, 29.6%; and Males, 21.8%). Single-peptide matched proteins accounted only for 4.8% of all proteins identified. Protein quantifications were based on the method proposed by Schirle *et al*. [7], where protein abundances were represented by the ratio of all MASCOT protein scores including redundant peptide scores versus the total number of MS/MS spectra of the sample where the protein detected, and was indicated as the following equation:

$$E = \frac{\sum PS}{N} \times 1000$$

E: Expression level of a protein; PS: peptide scores; N: total MS/MS spectra of the specific samples. It is known that the number of sequenced peptides/sequence coverage is in accordance with the MS signal intensity and the amount of proteins, as is the MASCOT score [7]. The combined MASCOT score was used as the measure for abundance taking into consideration the fact that the total number of unique peptides of a

given protein was correlated with the mass peak width, and intensity (peak height) [8].

References

1. Oaks JA, Cain GD, Mower DA, Raj RK (1983) Comparison of calcium, freeze-thaw, and triton X-100 tegumental disruption/recovery techniques applied to *schistosoma mansoni*. J. Parasitol. 69: 519-533.

2. Ozkan ED, Lee FS, Ueda T (1997) A protein factor that inhibits ATP-dependent glutamate and gamma -aminobutyric acid accumulation into synaptic vesicles: Purification and initial characterization. Proc Natl Acad Sci U S A. 94: 4137-4142.

3. Picoult-Newberg L, et al. (1999) Mining SNPs from EST databases. Genome Res. 9: 167-174.

4. Batley J, Barker G, O'Sullivan H, Edwards KJ, Edwards D (2003) Mining for single nucleotide polymorphisms and insertions/deletions in maize expressed sequence tag data. Plant Physiol. 132: 84-91.

5. Shevchenko A, Wilm M, Vorm O, Mann M (1996) Mass spectrometric sequencing of proteins from silver-stained polyacrylamide gels. Anal. Chem. 68: 850-858.

6. Resing KA, Meyer-Arendt K, Mendoza AM, Aveline-Wolf LD, Jonscher KR, et al. (2004) Improving reproducibility and sensitivity in identifying human proteins by shotgun proteomics. Anal. Chem. 76: 3556-3568.

7. Schirle M, Heurtier M-A, Kuster B (2003) Profiling core proteomes of human cell lines by one-dimensional PAGE and liquid chromatography-tandem mass spectrometry. Mol Cell Proteomics. 2: 1297-1305.

8. Lasonder E, Ishihama Y, Andersen JS, Vermunt AM, Pain A, et al. (2002) Analysis of

the *Plasmodium falciparum* proteome by high-accuracy mass spectrometry. Nature 419: 537-542.
