# Supplementary material for: New Perspectives on Host-Parasite Interplay by Comparative Transcriptomic and Proteomic Analyses of Schistosoma japonicum
Source: PLoS Pathog. 2006 Apr 14;2(4):e29. doi: 10.1371/journal.ppat.0020029 (PMC1435792; doi:10.1371/journal.ppat.0020029)

Figure. S1. Schematic Representation of the Strategy for the Integrated *S. japonicum* Transcriptome and Proteome Analyses

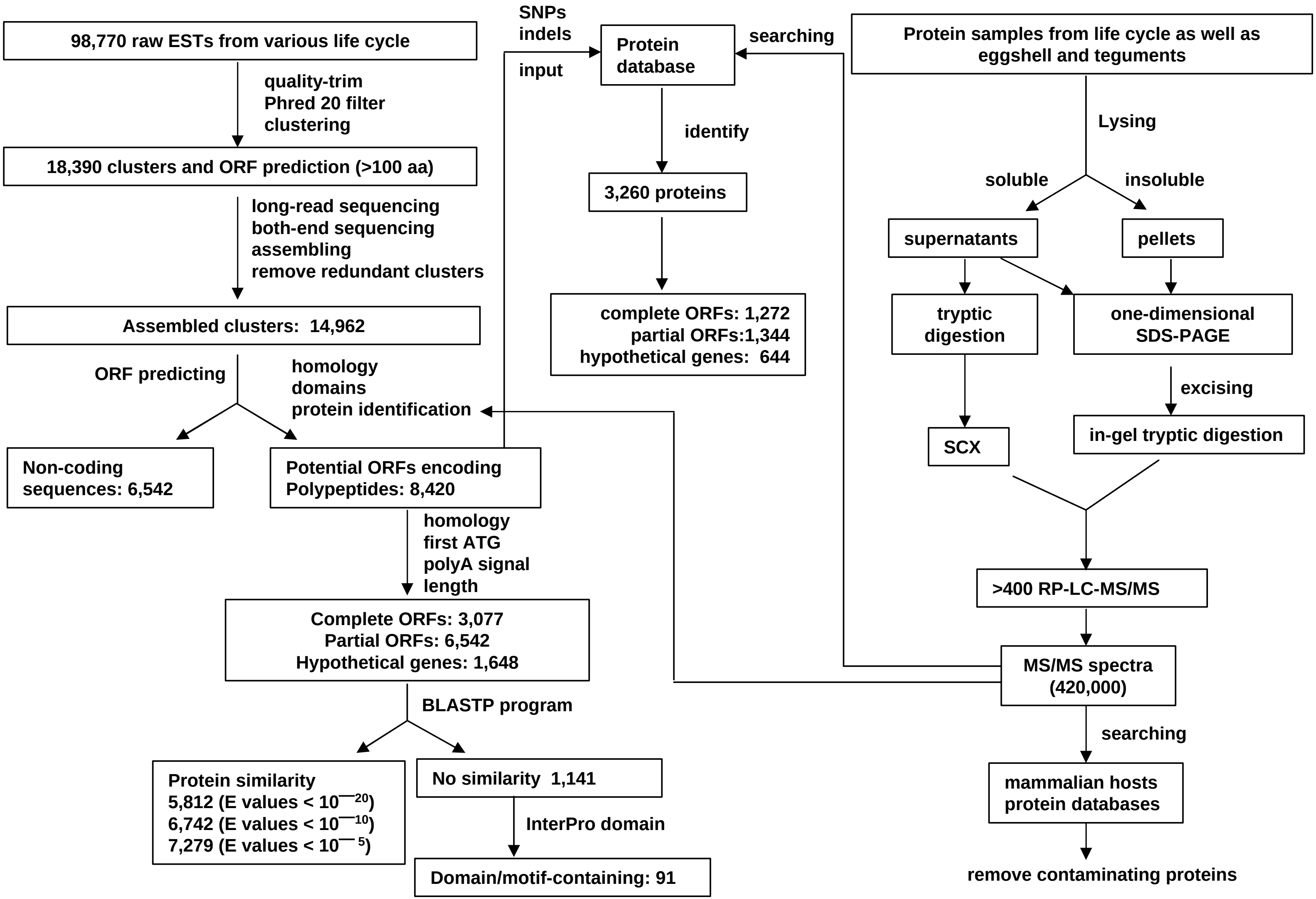

Supplement: Supplementary file 2 [file ppat.0020029.sg001.pdf]
