# Supplementary material for: New Perspectives on Host-Parasite Interplay by Comparative Transcriptomic and Proteomic Analyses of Schistosoma japonicum
Source: PLoS Pathog. 2006 Apr 14;2(4):e29. doi: 10.1371/journal.ppat.0020029 (PMC1435792; doi:10.1371/journal.ppat.0020029)
Supplement: Supplementary file 4 [file ppat.0020029.sg003.pdf]

Figure S3. Predicted functions of *S. japonicum* CDSs and proteins based on gene ontology (GO)

a

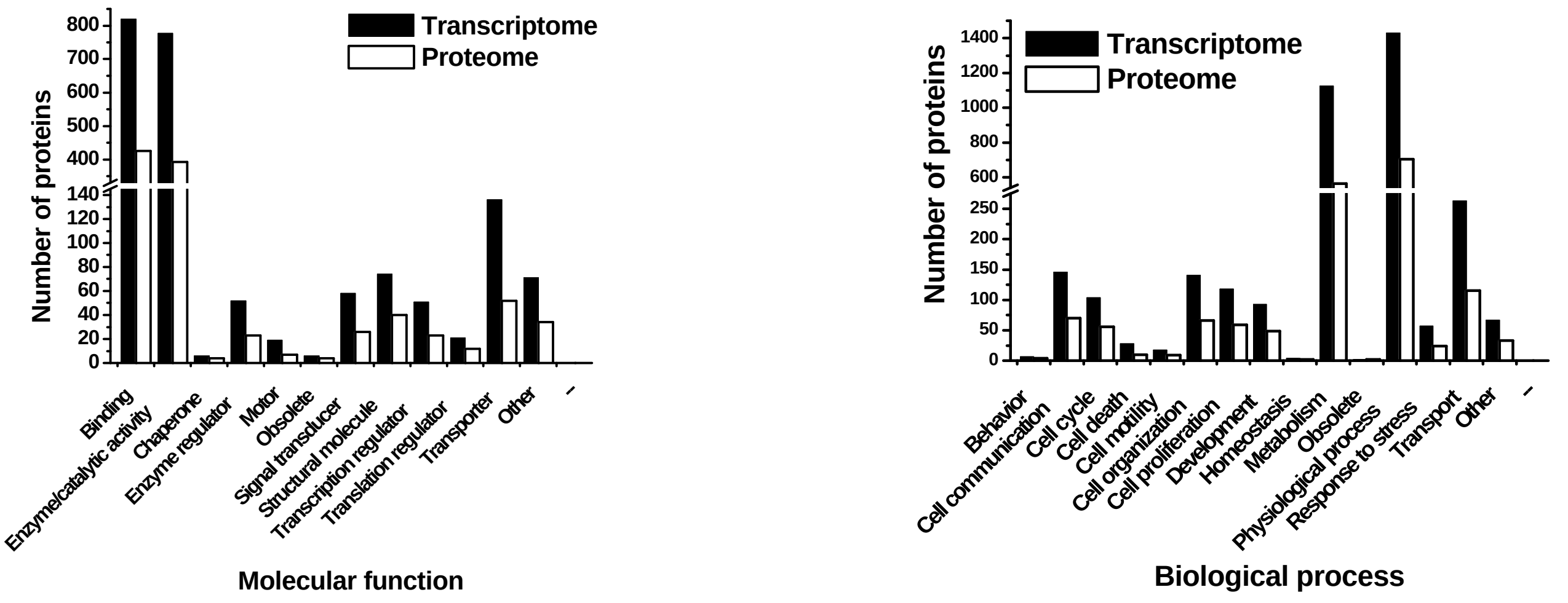

b

GO/function	C	S	A	E	Mi
Binding	173	190	217	215	136
Chaperone	2	2	2	3	3
Enzyme regulator	6	6	10	10	9
Enzyme/catalytic activity	145	158	166	164	120
Motor	3	4	3	3	2
Obsolete	2	2	2	3	3
Signal transducer	7	9	12	9	6
Structural molecule	17	21	25	18	14
Transcription regulator	11	10	10	10	5
Translation regulator	3	5	7	5	4
Transporter	14	14	24	18	13
Other	10	9	12	15	10
Total	393	430	490	473	325

GO/process	C	S	A	E	Mi
Behavior	2	1	1	1	1
Cell communication	23	22	29	26	21
Cell cycle	22	25	26	23	14
Cell death	2	5	5	3	5
Cell motility	6	4	4	2	3
Cell organization	34	31	27	24	29
Cell proliferation	23	25	27	25	15
Development	12	19	16	20	13
Homeostasis	0	1	1	0	0
Metabolism	247	237	250	279	193
Obsolete	1	1	1	1	2
Physiological process	314	296	314	343	236
Response to stress	13	11	13	16	7
Transport	51	49	48	53	43
Other	9	11	17	15	12
Total	759	738	779	831	594
