# Supplementary material for: New Perspectives on Host-Parasite Interplay by Comparative Transcriptomic and Proteomic Analyses of Schistosoma japonicum
Source: PLoS Pathog. 2006 Apr 14;2(4):e29. doi: 10.1371/journal.ppat.0020029 (PMC1435792; doi:10.1371/journal.ppat.0020029)
Supplement: Supplementary file 5 [file ppat.0020029.sg004.pdf]

Figure S4. Correlation between transcriptomic and proteomic data of representative proteins among life cycle stages and sexes

Notes:

The expression levels of S. *japonicum* proteins were calculated according to the following formulas.

Protein: Expression= Σ peptide_score/Total_MSMS*1000

mRNA: Expression=Cluster_ESTs/Library_ESTs*10000

C, cercaria; S, hepatic schistosomula; A, adult; E, egg; Mi, micidia.

The color scale indications are listed at the bottom of this table.

Cercaria enriched proteins

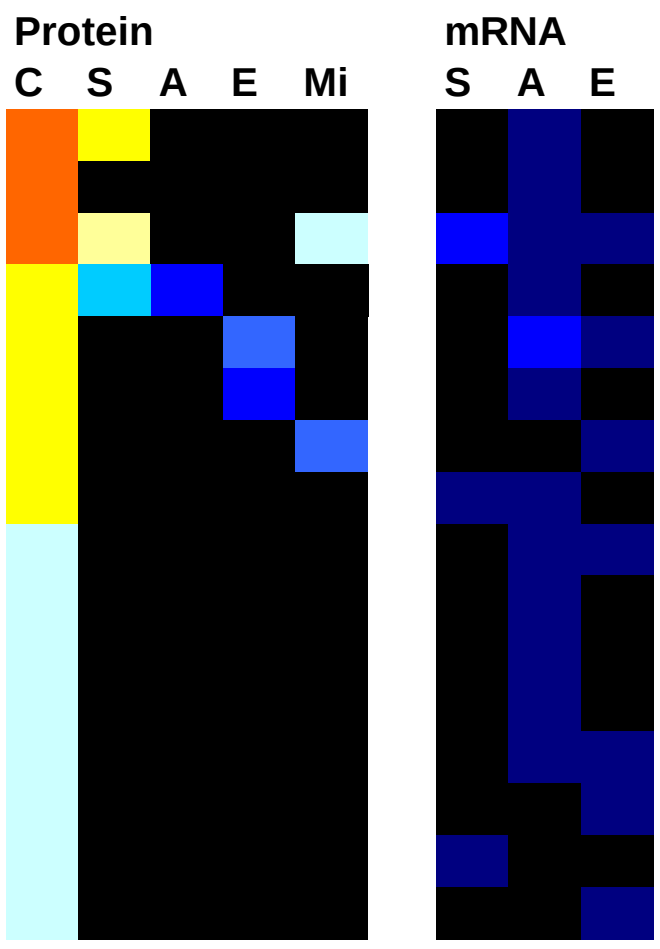

AY915076	Similar to EAA51569-hypothetical protein MG03164.4
AY915759	Similar to L36833 phosphoglycerate kinase in *S. mansoni*
AY809768	Similar to gelsolin (*Suberites domuncula*)
AY815594	Hypothetical protein
AY814661	Similar to zgc:86647 (*Danio rerio*)
AY812855	Similar to ENSANGP00000012999 (*Anopheles gambiae*)
AY811679	Similar to U86673 tegumental antigen Sm20
AY814087	Similar to B54211 H+-transporting two-sector ATPase
AY810624	Similar to glycyl-tRNA synthetase (human)
AY915770	Hypothetical protein
AY809437	Hypothetical protein
AY808651	Hypothetical protein
AY915337	Similar to AAH55791-Unknown
AY915772	Hypothetical protein
AY811327	Similar to XP_394506-similar to finger protein - frog
AY815112	Hypothetical protein

Hepatic schistosomula enriched proteins

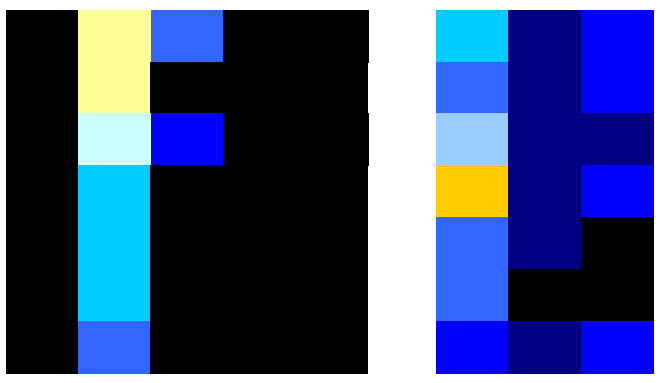

AAP06189	Similar to L78668 60S ribosomal protein L5A
AY815283	Similar to XM_084887 similar to hypothetical protein CGI-131 in human
AY815425	Similar to Hypothetical protein CBG11196 (*C. briggsae*)
AY811240	Similar to XP_392864-similar to lethal giant larvae homolog 1
AY814116	Similar to lipopolysaccharide-induced TNF factor (*Gallus gallus*)
AY808316	Similar to DX11 (fly)
AY813059	Similar to autophagy Apg3p/Aut1p-like (mouse)

Adult enriched proteins

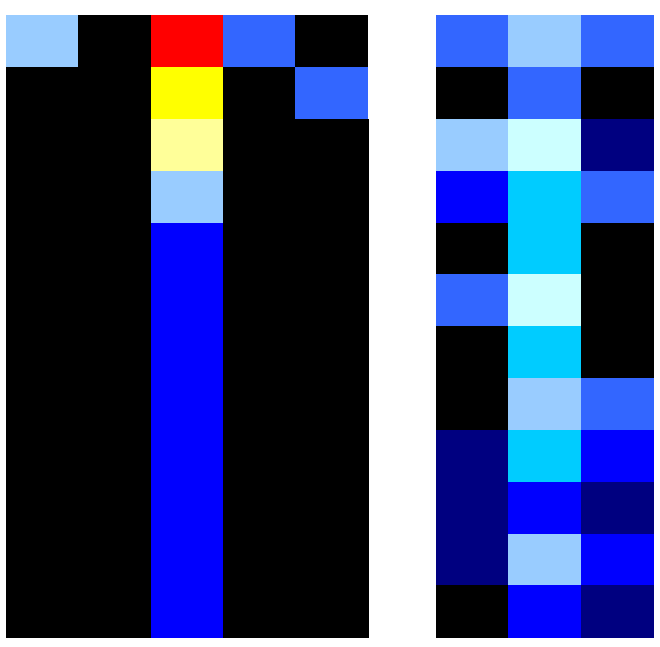

AY815599	Similar to AF207818 putative gap junction protein pannexin in *Clione limacina*
AY812195	Extracellular superoxide dismutase
AAP05942	Hypothetical protein
AAK57435	Serine protease inhibitor serpin
AAP05897	Similar to eggshell protein in *S. mansoni*
AAP06392	Hypothetical protein, putative Saposin type B
AY813550	Similar to AAB86571 protein-unknown (*S. mansoni*)
AY814927	Similar to CG7364-PA (fly)
AY814594	Similar to CG6323-PA (fly)
AY815435	Similar to NP_956429-hypothetical protein MGC55505 (*Danio rerio*)
AY815149	Similar to CG5510-PA (fly)
AY812786	Similar to AAP06353-similar to NM_000308 beta-galactosidase 2

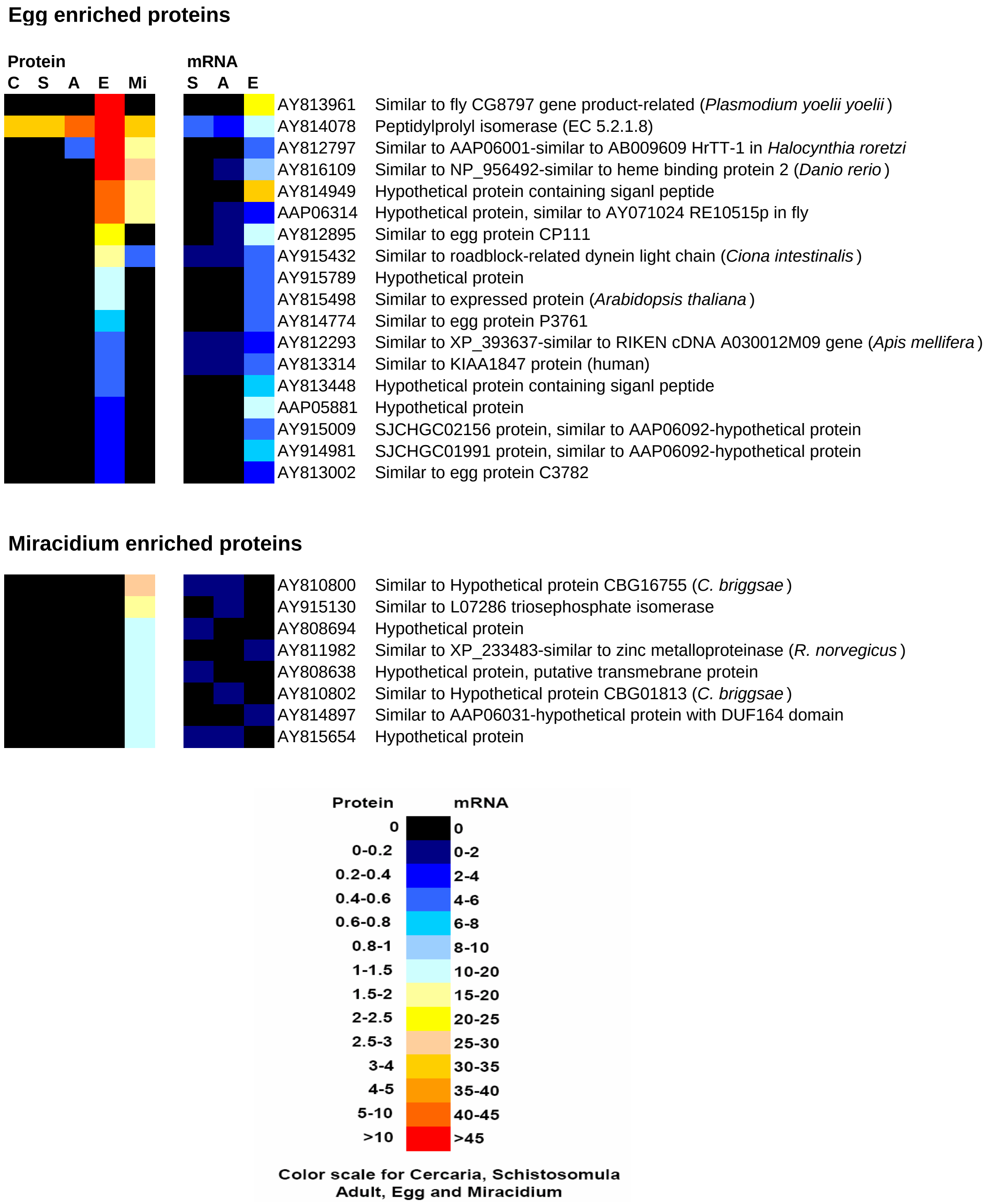
Egg enriched proteins
Protein
C S A E Mi
mRNA
S A E
AY813961 Similar to fly CG8797 gene product-related (*Plasmodium yoelii yoelii*)
AY814078 Peptidylprolyl isomerase (EC 5.2.1.8)
AY812797 Similar to AAP06001-similar to AB009609 HrTT-1 in *Halocynthia roretzi*
AY816109 Similar to NP_956492-similar to heme binding protein 2 (*Danio rerio*)
AY814949 Hypothetical protein containing siganl peptide
AAP06314 Hypothetical protein, similar to AY071024 RE10515p in fly
AY812895 Similar to egg protein CP111
AY915432 Similar to roadblock-related dynein light chain (*Ciona intestinalis*)
AY915789 Hypothetical protein
AY815498 Similar to expressed protein (*Arabidopsis thaliana*)
AY814774 Similar to egg protein P3761
AY812293 Similar to XP_393637-similar to RIKEN cDNA A030012M09 gene (*Apis mellifera*)
AY813314 Similar to KIAA1847 protein (human)
AY813448 Hypothetical protein containing siganl peptide
AAP05881 Hypothetical protein
AY915009 SJCHGC02156 protein, similar to AAP06092-hypothetical protein
AY914981 SJCHGC01991 protein, similar to AAP06092-hypothetical protein
AY813002 Similar to egg protein C3782
Miracidium enriched proteins
AY810800 Similar to Hypothetical protein CBG16755 (*C. briggsae*)
AY915130 Similar to L07286 triosephosphate isomerase
AY808694 Hypothetical protein
AY811982 Similar to XP_233483-similar to zinc metalloproteinase (*R. norvegicus*)
AY808638 Hypothetical protein, putative transmembrane protein
AY810802 Similar to Hypothetical protein CBG01813 (*C. briggsae*)
AY814897 Similar to AAP06031-hypothetical protein with DUF164 domain
AY815654 Hypothetical protein
Protein
mRNA
0
0
0-0.2
0-2
0.2-0.4
2-4
0.4-0.6
4-6
0.6-0.8
6-8
0.8-1
8-10
1-1.5
10-20
1.5-2
15-20
2-2.5
20-25
2.5-3
25-30
3-4
30-35
4-5
35-40
5-10
40-45
>10
>45
Color scale for Cercaria, Schistosomula
Adult, Egg and Miracidium

Female enriched proteins

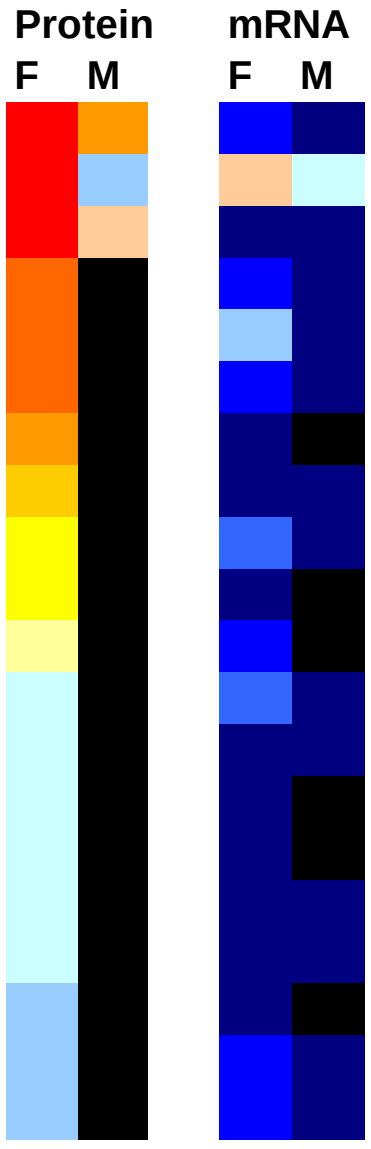

AY813753	Similar to dolichyl-diphosphooligosaccharide-protein glycosyltransferase
AY814058	Elongation factor 1-alpha
AY814062	Similar to Ribosomal protein L4 (*R. norvegicus*)
AY812195	Extracellular superoxide dismutase
AAP05942	Hypothetical protein
AY815168	Similar to CG4087-PA (fly)
AY809963	Similar to ribophorin II (mouse)
AAP06462	Similar to ribosomal protein S3 in mouse
AY815389	Immunophilin
AY815049	Similar to rAsp f 8 (*Aspergillus fumigatus*)
AY812921	Hypothetical protein
AAP05897	Similar to Z27402 eggshell protein in S. mansoni
AAP06276	Similar to AY044241 glutamine synthetase
AY810269	Cct5-prov protein
AY814137	Similar to BAB32325-unnamed protein product (mouse)
AY810713	Glutaminyl-tRNA synthetase
AAP05886	Similar to AJ279092 cathepsin L1 in *Fasciola hepatica*
AY815492	Similar to female-specific 800 protein (*S. mansoni*)
AY816104	Similar to annexin B13a in *Bombyx mori*
AY811026	Protein disulfide isomerase homology

Male enriched proteins

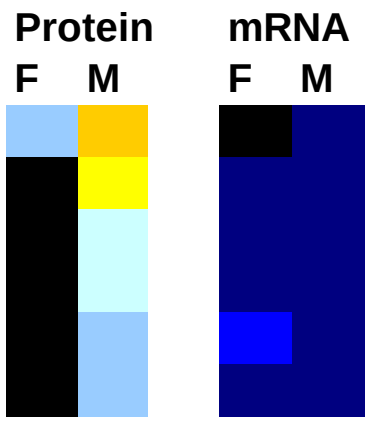

AAN39279	Gynecophoral canal protein
AY815855	Similar to F-box only protein 9
AY814931	Similar to calcium binding atopy-related autoantigen 1
AAP06409.	Similar to BC003643 RIKEN cDNA 3222401G21 gene in human
AAP06180	Similar to NM_065417 casein Kinase I in *C. elegans*
AY815961	Hypothetical protein CBG13013

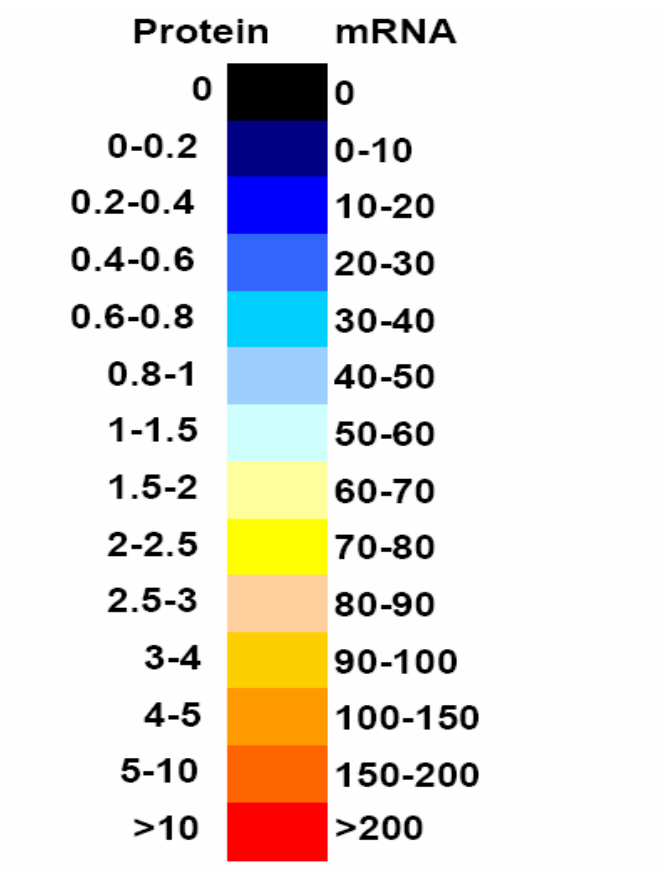

Color scale for Female and male
