# Supplementary material for: New Perspectives on Host-Parasite Interplay by Comparative Transcriptomic and Proteomic Analyses of Schistosoma japonicum
Source: PLoS Pathog. 2006 Apr 14;2(4):e29. doi: 10.1371/journal.ppat.0020029 (PMC1435792; doi:10.1371/journal.ppat.0020029)

Figure S5. Verification of SNPs by DNA sequencing

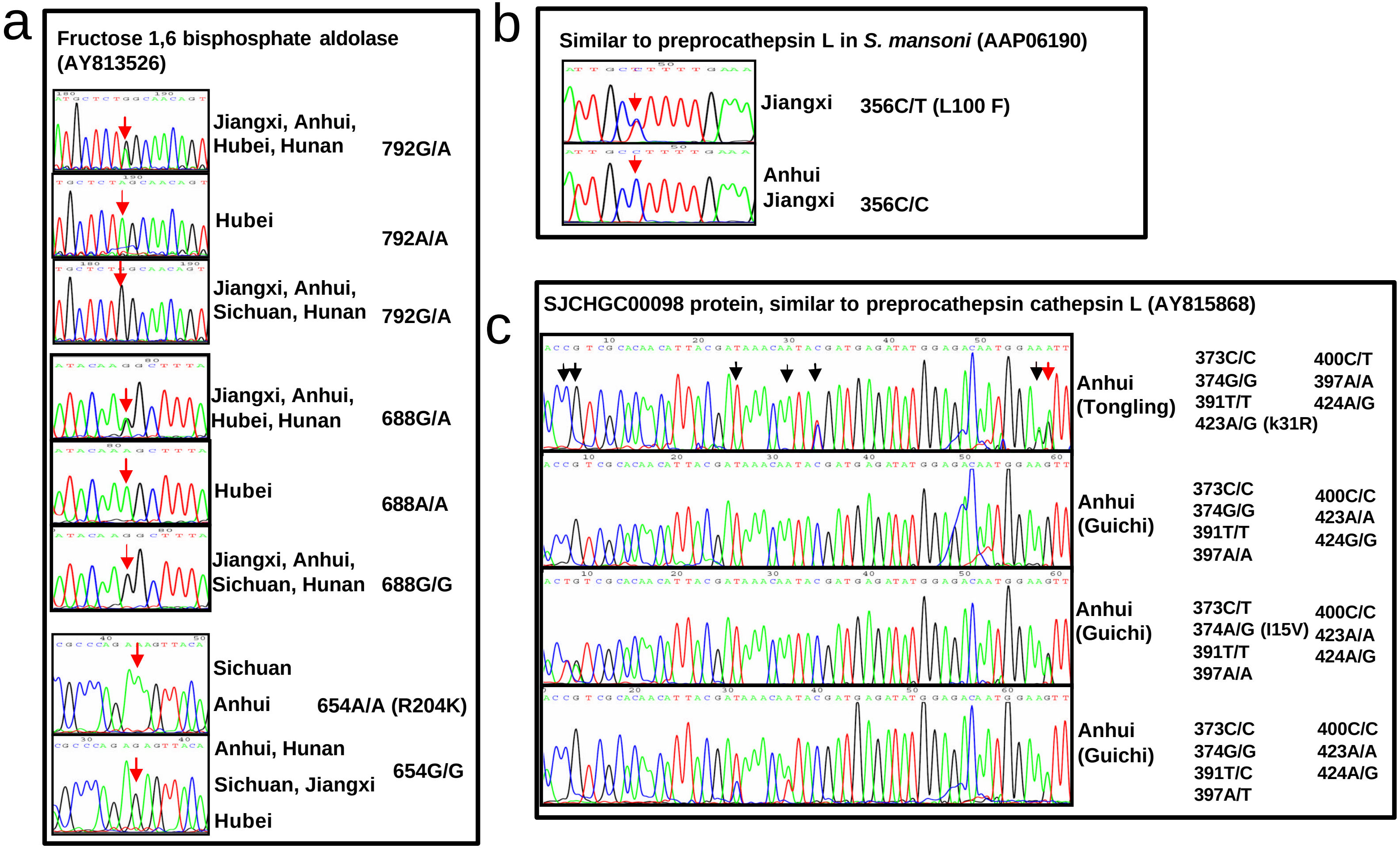

Supplement: Supplementary file 6 [file ppat.0020029.sg005.pdf]
